# Translation and multi-site validation of the ‘Pediatric Complex Care Needs Assessment Scale*’* (ACCAPED) from Italian to English

**DOI:** 10.1186/s12904-026-02031-1

**Published:** 2026-03-03

**Authors:** Kim Sadler, Steven Callaghan, Aza AlSawafi, Spandana Rayala, Wejdan Alghamdi, Anna Marinetto, Pierina Lazzarin, Saadiya Khan, Khaled AlGhamdi, Fawad Ahmad, Raghad Alhuthil, Hamad Hussain Alyami, Tala Al-Dabbous, Nidhal AlHarrasi, Maryam AlBattashi

**Affiliations:** 1 Cancer Center of Excellence, King Faisal Specialist Hospital & Research Center, P.O. 3354, Riyadh, 11211 Saudi Arabia; 2 Child Health Department, The Royal Hospital, Muscat, Oman; 3 Bayt Abdullah Children’s Hospice, Kuwait City, Kuwait; 4 Department of Medicine, University of Padua, Padua, Italy; 5 Healthcare Profession Department, Padua University Hospital, Padua, Italy; 6 Department of Women’s and Children’s Health, University of Padua, Padua, Italy; 7 Pediatric Hematology-Oncology Department, King Faisal Specialist Hospital & Research Center, Riyadh, Saudi Arabia; 8 Pediatrics Department, King Faisal Specialist Hospital & Research Center, Riyadh, Saudi Arabia; 9 Al Faisal University, Riyadh, Saudi Arabia; 10 Women and Pediatric Cancer Excellence King Faisal Specialist Hospital & Research Center, Riyadh, Saudi Arabia

**Keywords:** Complex care, Pediatrics, Pediatric palliative care, Scale, Assessment, ACCAPED, Palliative care

## Abstract

**Background:**

Few tools exist to assess the palliative care needs of children with serious illnesses. One such tool is the Pediatric Complex Care Needs Assessment scale (ACCAPED), developed by a group of pediatric palliative care experts in Italy. It evaluates care complexity across 11 clinical domains to categorize palliative care needs into three levels: low, moderate, and high.

**Aim:**

This study aimed to translate and validate the Pediatric Complex Care Needs Assessment scale (ACCAPED) from Italian into English.

**Design:**

The methodology included (1) translation, (2) expert panel validation, (3) end-user validation, (4) expert panel review, and (5) multi-site validation with a sample of children with various conditions and needs from Saudi Arabia, Kuwait, and Oman (N = 199).

**Results:**

During the translation and validation process, optimization strategies were employed to improve the scale’s psychometric properties, including adjusting the weighting of the clinical needs domain, modifying the complexity category thresholds, adding three items, and removing 16 items, thereby enhancing the scale’s construct validity. Using a regression model, factors such as mobilization, pain, instability, and skin integrity were identified as significant influences on expert judgments of patient complexity. Like the original, the translated English version contains 11 domains.

**Conclusion:**

The English Modified-*ACCAPED* scale appears to be a practical tool for screening for palliative care needs in children with potentially life-limiting conditions and for guiding them early in their disease trajectory toward appropriate resources. However, future research is recommended to strengthen its psychometric properties.

**Supplementary Information:**

The online version contains supplementary material available at 10.1186/s12904-026-02031-1.

## Introduction

Palliative care (PC) for children with life-limiting or life-threatening conditions aims to enhance quality of life by supporting symptom management [1], psychosocial care [2], and family-centered decision-making [3]. When introduced early—alongside disease-modifying treatments—it promotes goal-based planning and continuity of care [4]. Many eligible children are also categorized as Children with Medical Complexity (CMC), typically with non-malignant neurological, congenital, or perinatal conditions. [5–6] They experience significant functional limitations and often rely on medical technologies [7], resulting in high service utilization and a substantial burden on families. Medical advances have increased survival but also increased exposure to intensive interventions that may affect quality of life.

PC may be delivered across three levels—primary, generalist, and specialized care—with the required level evolving over a child’s illness. All healthcare providers caring for children eligible for PC should possess basic palliative care competencies. At the same time, teams with frequent exposure to serious illness, such as oncology teams, are expected to develop more advanced skills. Specialized PC (subspecialty), delivered by trained multidisciplinary teams, plays a crucial role in managing refractory symptoms, complex decision-making, conflicts around goals of care, and significant emotional distress. Despite clear benefits, access to PC services remains limited. Barriers include restricted medication access (e.g., opioids), inadequate provider training [8], workforce shortages [9], and persistent misconceptions that conflate PC with end-of-life care or “giving up” [9–11]. When services are available, late referrals to specialized PC are common; for example, only 44.2% of children with advanced cancer in one U.S. study were referred to specialized PC [12], often due to provider optimism about prognosis or fear of reducing hope [13].

Another significant barrier is the difficulty in early identification of children who could benefit, including the lack of validated screening tools for detecting PC needs. Some current options include the Paediatric Palliative Screening Scale (PaPaS) and the Pediatric Complex Care Needs Assessment Scale (ACCAPED) [14–16]. Both help clinicians determine which children require PC services and which can be managed with less-intensive, community-based care (primary pediatric care) or need specialized PC.

### Paediatric Palliative Screening Scale (PaPaS)

The PaPaS scale is a brief, multidimensional screening tool that assesses five key aspects relevant to palliative care needs: disease progression and its effects on daily life; expected outcomes and the burden of disease-specific treatments; symptom severity; patients’, parents/caregivers’, and healthcare providers’ care preferences; and estimated life expectancy. Based on the final score, the child is categorized as either “no PC needed,” “PC to be considered,” or “PC needed.” It has been validated in English and used in several studies.

### Pediatric Complex Care Needs Assessment Scale (ACCAPED)

The Pediatric Complex Care Needs Assessment Scale [original version: Accertamento dei bisogni Clinico-Assistenziali Complessi in Pediatria (It.)] assesses the clinical needs of children with life-limiting or life-threatening illnesses [15]. It allows for a more comprehensive evaluation across 11 clinical domains: breathing, feeding, seizures/altered consciousness, skin integrity, mobility, communication, rest and sleep, continence and evacuation, medication administration, and pain. It also includes the surprise question: ‘‘Would you be surprised if this child died within the next 12 months? [17–18] The final score classifies PC needs as low, moderate, or high complexity, indicating the recommended level of PC expertise (Table 1). Before this study, it had not been validated in English [15]. 

**Table 1:** Palliative care expertise level suggested based on the English modified *ACCAPED* scale final score

Level of Care Complexity	Adjusted Cutoff Scores	PC Expertise Level Recommended
Low	≤ 27	Level 1 Primary Palliative Care (Approach) Primary care, community care, local healthcare facilities, Family Medicine, General Pediatrics *PC Provided by first-line healthcare providers or Primary medical teams (e.g.*,* Pediatricians).*
Moderate	28–64	Level 2 General Complex/Palliative Care Secondary care, hospital, medical specialties *PC Provided by healthcare with additional training in complex/palliative care and palliative care specialists (as needed)(e.g.*,* oncologists). Consider the input of Allied Healthcare providers (e.g.*,* occupational therapists*,* physiotherapists*,* rehabilitation centers).*
High	≥ 65	Level 3 Specialized Complex/Palliative Care (subspecialty) Tertiary care, hospitals, hospice, palliative care home *PC Provided by Complex/Palliative Care specialists and medical subspecialties (as needed)*.

This study aimed to translate and validate the ACCAPED scale into English to help identify children who might benefit from PC services earlier and to evaluate the level of PC expertise needed.

## Methods

**Fig. 1:**
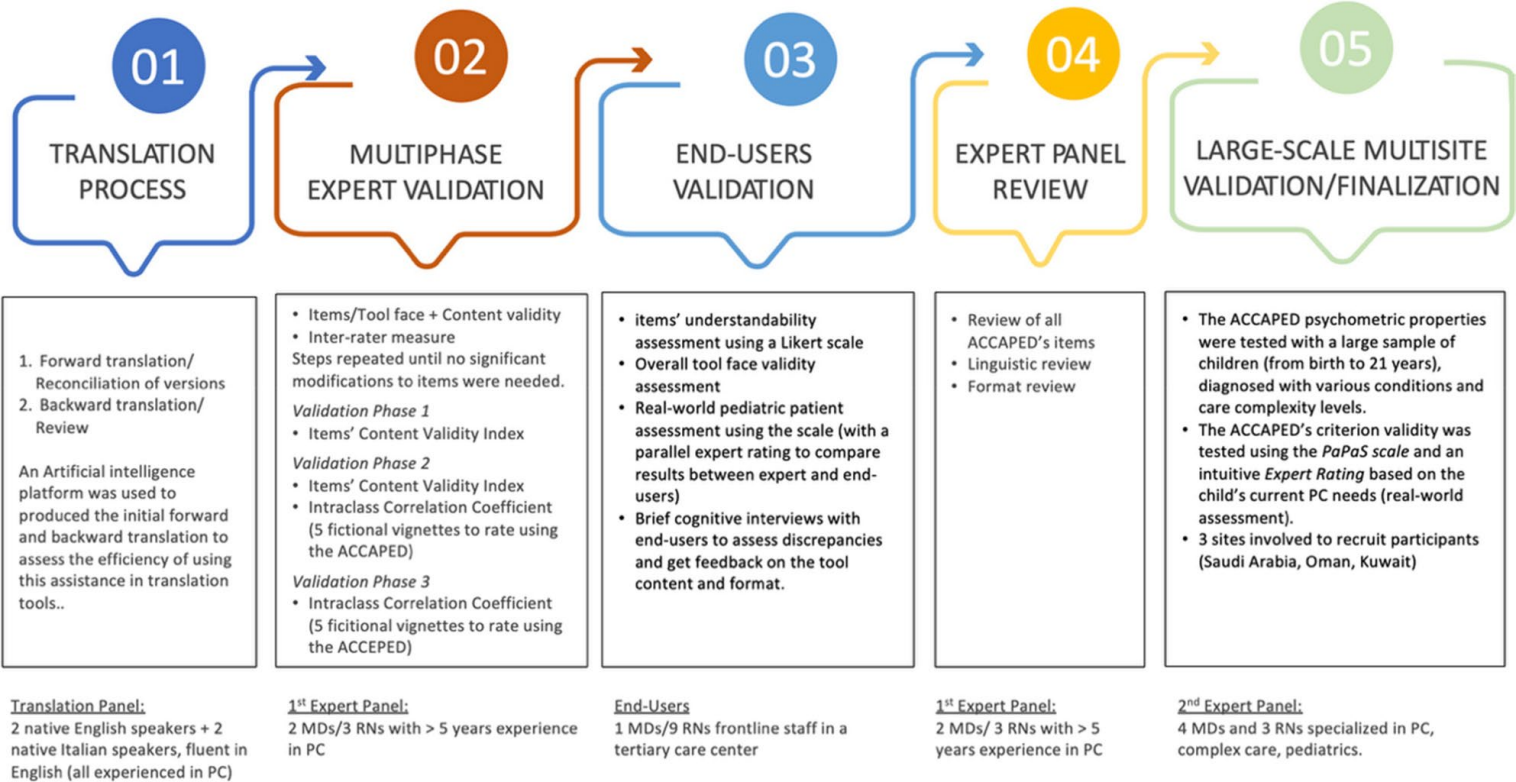
ACCAPED scale translation and multi-phase validation process

The process followed the methodological approach recommended by the World Health Organization’s guidelines for translating and adapting instruments [19]. The approach involved five steps: (1) translation, (2) multi-phase expert validation, (3) end-user validation, (4) expert panel review, and (5) large-scale multisite validation and finalization (Fig. 1). However, this method was slightly modified to evaluate the benefits of using artificial intelligence to enhance translation efficiency.

### Preparation

Permission to translate and adapt the tool was granted by the original developers, who are also part of this study (P.L./A.M.). The translation panel included two native English speakers and two of the original authors (P.L./A.M.), who are native Italian speakers fluent in English. All had experience in palliative and/or pediatric care.

### Step 1: translation

#### Forward translation and reconciliation

A forward translation of the entire ACCAPED scale from Italian to English was performed using an AI platform [*ChatGPT*, 2023]. Afterwards, the translation panel members independently evaluated the linguistic quality and clarity of the translation, then reached consensus to unify their versions.

#### Backward translation and review

The same AI platform was used for a backward translation into Italian. The panel reviewed this version against the original to identify any inconsistencies in the item’s meaning.

### Step 2: multi-phase expert validation

An expert panel for Step 2 (Table 2) was composed of five nurses and physicians, each with more than 5 years of experience in Pediatric Complex/PC and publications in the field. This step focused on assessing the face and content validity of the English translation. Face validity assesses how well an item’s content fits the context in which the tool will be used [20]. It considers the appropriateness, sensibility, and relevance of the item. Content validity examines how comprehensively the items cover all aspects of the construct being measured [20]. The process was repeated until no significant modifications were needed for the tool’s items.

**Table 2:** Expert panels and end-users sample characteristics and work experience

Characteristics	Expert Panel 1* (*n* = 5)		Expert Panel 2** (*n* = 7)		End Users (*n* = 10)	
	*n*	%	*n*	%	*n*	%
Occupation						
Physician	2	40	4	57	1	10
Nurse	3	60	3	43	9	90
Work Experience						
< 5 years	0	0	1	14	2	20
5–10 years	1	20	1	14	2	20
> 10 years	4	80	5	72	6	60
Population Served						
Malignancy	0	0	1	14	4	40
Non-malignancy	0	0	2	28	3	30
Both	5	100	4	58	3	30

* Experts included in the *Step 3: Multi-Phase Expert Validation*

** Experts included in the *Large-Scale Multisite Validation* (Expert Rating)

### Phase 1

Using a 5-point Likert scale, all items were rated for their value in assessing the 11 domain constructs of the ACCAPED scale (1 = low value to 5 = high value). This enabled calculation of a Content Validity Index at both the item level (I-CVI) and the scale level (S-CVI/Ave, S-CVI/UA). A Content Validity Index (CVI) measures expert consensus regarding the relevance of items. The I-CVI assesses this agreement for each item, while S-CVI/Ave and S-CVI/UA summarize agreement across the entire scale, including only items with complete expert agreement. For scoring, ratings of 1–3 were assigned a value of 0 (low), and ratings of 4 and 5 received a value of 1 (high). According to the methodological article by Shi et al. [21], I-CVI scores of 0.78 or higher, along with S-CVI/UA and S-CVI/Ave scores of 0.8 and 0.9 or above, respectively, are considered excellent. Based on these metrics, the items were removed, adjusted, maintained, or added through consensus with the expert panel.

### Phase 2

After completion of the previous phase, experts repeated the same task using the reviewed ACCAPED scale. They were also asked to rate five written fictional clinical vignettes depicting children with various conditions and care needs using the tool (Refer to Table 3 for an example).

**Table 3:** Example of fictional clinical vignette

Salma, who is 3 years old, was diagnosed with a neurodegenerative disorder accompanied by significant global developmental delay. Recently, she has experienced a faster regression in several milestones. She can no longer sit for a year or grasp objects. She has never produced any vocalizations or expressive language. She also suffers from significant spasticity, which is well-controlled with medication. Her other medical issues include a history of gastrointestinal reflux, currently managed with daily omeprazole. She has a seizure disorder with partial seizures occurring several times a week, each lasting a few minutes, and is on three anticonvulsant medications. These medications require frequent adjustments. For the past six months, she has been fed exclusively through a PEG tube, and all her medications are also administered via this route by her caregiver. She tolerates the feeding well, and her weight is adequate. Her breathing is normal on room air. There are no skin issues. She is incontinent of both urine and stool, using a diaper. She has difficulty falling asleep at night and sleeps most of the day.	

Using an absolute agreement definition, single-measure and average-measure Intraclass Correlation Coefficients (ICCs) were calculated to provide a more complete understanding of the translated version’s reliability. The single-measure ICC indicates how dependable one rater’s score would be in everyday practice. The average-measure ICC reflects the reliability when ratings from multiple assessors are combined.

### Phase 3

Experts were asked to evaluate the same five fictional written vignettes using the ACCAPED scale reviewed in the previous phase. Single-measure and average-measure Intraclass Correlation Coefficients (ICCs) were also calculated.

### Step 3: end-user validation

Ultimately, when validating a tool, it must be tested with a group of the intended users, the ‘end-users.’ A group of 10 nurses and physicians was recruited through convenience sampling to (1) review the ACCAPED scale’s items to assess their understandability (linguistic clarity) using a 5-point Likert scale (1 = very easy to 5 = very difficult), (2) comment on the tool’s face validity, and (3) use the scale to score the care complexity of a real-world pediatric patient recruited through convenience sampling in a tertiary care center in Saudi Arabia (Table 2). This patient was also independently evaluated by a researcher to allow for score comparison. Additionally, after completing these tasks, a brief cognitive interview was conducted to review the end-users’ ratings. The “think-aloud” method was used, which enables participants to share their thoughts spontaneously and openly when encountering or hearing a question [22]. They were asked to explain their reasoning if their scores differed from the expert’s and to provide feedback on the tool’s overall content and format. Two researchers (K.S. / S.C.), part of the expert panel, conducted these brief interviews. The researchers summarized and documented the end-users’ responses themselves. Based on this feedback and the expert panel’s review, adjustments were made to the tool’s content and format.

### Step 4: expert panel review

The original expert panel reviewed all quantitative and qualitative data collected during the validation process. The tool was also checked for linguistic errors and its format finalized.

### Step 5: large-scale multisite validation

The tool’s criterion validity and reliability were further evaluated to assess its ability to measure the care complexity of pediatric patients’ needs across a large sample of children and adolescents with various medical conditions and levels of need (birth to 21 years old). Participants were recruited through convenience sampling from inpatient and outpatient settings at three centers in Saudi Arabia, Oman, and Kuwait. The ideal sample size for this part of the tool validation remains a topic of debate among researchers. For this study, as outlined in Aithal and Aithal’s article [23], a sample of 200 participants was considered appropriate. Two measures of concurrent validity were used: (1) the *PaPaS* scale and (2) an intuitive *Expert Rating* of the PC needs’ complexity (Low, Moderate, High), based on the level of PC expertise required to manage the child’s current needs. A Cohen’s kappa test was used to assess agreement between the different measures, and an ordinal logistic regression examined the extent to which the ACCAPED scale’s domains predicted the *Expert Rating.* Data collection and *Expert Ratings* for this phase were conducted by a different panel consisting of seven experts, including four physicians with experience in pediatrics, complex care, palliative care, and/or hematology/oncology; two advanced clinical specialist nurses; and one pediatric clinical specialist nurse with experience in either palliative care or hematology/oncology (Table 2).

### Ethical considerations

This study received ethics approval from the Institutional Review Boards of King Faisal Specialist Hospital & Research Center, Saudi Arabia (Reference #2231292), the Royal Hospital, Oman (MoH/CSR/25/28758), and the Bayt Abdallah Hospice, Kuwait (MoH 2025/2820). Informed consent was obtained from all participating healthcare providers and the children’s legal guardians, and minor assent was obtained from children aged 7 and above. Due to the minimal risks involved in the study, the IRBs granted a waiver of signed consent; verbal consent was documented in the children’s medical records. The research was conducted in adherence to the Declaration of Helsinki.

## Results

All modifications to the ACCAPED’s items during translation and validation are recorded in a table in the Supplementary Material.

### Step 1: translation

#### Forward translation/reconciliation

Following the forward translation and reconciliation, the format and content of selected items were found to be unclear and have been revised. In the *Breathing* domain, more precise distinctions among oxygen delivery methods were added to better illustrate their respective complexities (e.g., nasal cannula vs. non-rebreather mask vs. CPAP/BiPAP). In the *Nutrition/Hydration* domain, the original item on a *special diet* could refer either to its nutritional content or its consistency. It was changed to: “*Requires a special diet (e.g.*,* to improve caloric and nutrient intake)*,” to differentiate it from a dysphagia-specific diet, which involves greater risks and complexity. In the *Seizures/Altered Level of Consciousness* domain, items were adjusted to address overlap, with an adjective indicating seizure frequency added to each item. In the *Communication* domain, a new item was added to accurately cover the full spectrum: ‘*Vocalizations without obvious signification or non-verbal*,* with or without expressive language’*. Many children requiring PC, especially those with non-malignant conditions, display this feature, which complicates their assessment. Finally, in the *Pain/Irritability* domain, items were reformulated to be more objective by including references to pain/irritability intensity, frequency, and interventions needed for relief, better reflecting different levels of complexity.

#### Backward translation and review

No items were adjusted, demonstrating that the use of artificial intelligence was efficient.

### Step 2: multi-phase expert validation

The content validity index at the scale level for experts and end users is provided in Table 4. All changes to the ACCAPED, including added, modified, or removed items, are listed in Table 5.

**Table 4:** Content validity index at the scale level for expert and end user validation

	Expert Panel		End Users
	Phase 1 (*n* = 5)	Phase 2 (*n* = 5)	Phase 1 (*n* = 10)
S-CVI/Ave^1^	0.84	0.92	0.98
S-CVI/UA^2^	0.51	0.66	0.83
Total # items	81	74	66

^1^ Scale-Content Validity Index Average; S-CVI/Ave > 0.9 Excellent; ^2^ Proportion of items that were considered of high relevance by all the experts (scores of 4 or 5); (S-CVI/UA) > 0.8 excellent (Shi, Mo, Sun, 2012)

**Table 5:** Items removed and added during expert and end-user validation

Items	I-CVI	Action
After the Forward Translation		
Non-verbal (with or without expressive language).	-	Added
After the Expert Validation – Phase 1		
Increased susceptibility to lung infections.	0.25	Removed
Present an airway abnormality but can breathe independently.	0.25	Removed
The child is unstable due to uncontrollable seizures.	0.50	Removed
Compromised skin integrity, requires at least weekly clinical monitoring.	0.50	Removed
Reduced muscle tone that interferes with balance and/or movements.	0.75	Removed
Regular seizures or an altered level of consciousness up to a few times per month that respond well to therapy.	0.75	Removed
Communication is difficult to understand and interpret for all.	0.75	Removed
Has a tracheostomy that requires frequent suctioning.	1.00	Removed
Wound(s) requiring specialized dressing(s) multiple times per week, respond to treatment.	1.00	Removed
Continence care is problematic and requires regular interventions by a trained caregiver (e.g., rectal washouts).	1.00	Removed
Requires chest physiotherapy.	0.50	Adjusted
Possible foreseeable event(s) that may jeopardize the clinical stability but not necessarily cause death.	0.50	Adjusted
Uses a non-rebreather mask.	-	Added
Frequent seizures or altered levels of consciousness (more than 4 times a week) requiring complex interventions: drugs paired with safety protocols (e.g., oxygen administration, suctioning). 31	0.50	Maintained
Persistent seizures or altered level of consciousness (up to daily) refractory to therapy and posing a high risk to life (e.g., resistance to Midazolam, lorazepam, diazepam).	0.50	Maintained
Unexpected weight loss that requires interventions to ensure adequate nutrition and hydration.	0.6	Removed
Serious health risks during sleep are present and require immediate intervention (e.g., apnea, seizures, secretions requiring suctioning).	1.00	Removed
After the Expert Validation – Phase 2		
Dysphagia that requires interventions to ensure adequate nutrition and hydration.	0.6	Adjusted
Requires regular chest physiotherapy.	0.6	Maintained
After the Expert Validation – Phase 3		
Frequent sleep apnea episodes.	-	Removed
Presents breathing difficulties that pose a risk to life.	-	Removed
Seizures or altered levels of consciousness not responding to simple interventions and requiring complex interventions; drugs paired with safety protocols (e.g., oxygen administration, suctioning).	-	Removed
Moderate to severe pain requiring a combination of medication (e.g., regular gabapentin and acetaminophen or morphine as needed).	-	Removed
After the End Users Validation		
Clinically stable but foreseeable event(s) may jeopardize condition (e.g. high risk of sepsis).	0.8	Adjusted
Clinically unstable and progressing condition not responding to treatments. I would not be surprised if the child died within the next 12 months	0.8	Adjusted
Has a central line at home (e.g., PICC line, Port-o-Cath, Broviac, Gamcath).	-	Added

I-CVI : Content Validity Index at the Item level

### Phase 1

 A total of 11 items had an I-CVI score of 0.75 or lower and were reevaluated by the original expert panel. Seven of these were removed as considered too subjective, nonspecific, or overlapping with other items within the same domain (e.g., ‘Increased susceptibility to lung infections’). The item ‘Wound(s) requiring specialized dressing(s) multiple times per week, respond to treatment’ lacked distinction from other items in the same domain. The wording was slightly adjusted for two items; for instance, the term *‘regular’* was added to ‘*Requires chest physiotherapy*’ to distinguish it from healthy children who may only need this intervention occasionally during an acute infection and are thus not considered to have complex care needs at baseline. Two items with an I-CVI score of 0.50 in the *Seizures/Altered Level of Consciousness* domain were retained for retesting, as requested by the panel, after the item ‘The child is unstable due to uncontrollable seizures*’* was removed, aiming to clarify the range of complexity. Additionally, two items with an I-CVI of 1 were also removed because they overlapped with other items in the same domain, confusing scoring: ‘Continence care is problematic and requires regular interventions by a trained caregiver (e.g., rectal washouts)’ and ‘Wound(s) requiring specialized dressing(s) multiple times per week, respond to treatment’. The wording of some items in the *Seizures/Altered Level of Consciousness*, *Sleep/Rests*, and *Pain/Irritability* Assessment domains was slightly revised to enhance differentiation.

### Phase 2

Three items had an I-CVI of 0.60. One was removed: ‘Unexpected weight loss that requires interventions to ensure adequate nutrition and hydration’, due to a lack of agreement on the objective threshold and the time frame for this weight loss, and because it represented a consequence of feeding issues already included in the *Nutrition/Hydration* domain. The item ‘Requires a special diet to improve *caloric* and *nutrient* intake’ was revised to include clarification examples and specify that no dysphagia was present: “…(e.g., hypercaloric, low sodium, ketogenic diet). No dysphagia present.’ The last item with an I-CVI of 0.6, ‘Requires regular chest physiotherapy,’ was retained. The panel deemed it an essential item and decided to continue testing it before making any adjustments or removing it. Despite having an I-CVI of 1, an additional item in the *Sleep/Rest* domain was removed: ‘Serious health risks during sleep are present and require immediate intervention (e.g., apnea, seizures, secretions requiring suctioning)’ because these risks were already scored in other domains, such as *Breathing or Seizures/Altered Level of Consciousness*, and including it would duplicate the assessment and potentially inflate the final score.

The experts’ inter-rater reliability values for the clinical vignettes rating were 0.88 (95% CI: 0.83–0.92) (*p* < .001) for single measures and 0.97 (95% CI: 0.96–0.98) (*p* < .001) for average measures, showing strong agreement among raters.

### Phase 3

Since only four items were removed after the last version, no I-CVIs were calculated during Phase 3. Instead, experts independently reviewed all items, instructions, and the tool format one final time to identify any potential oversights. By consensus, four items were removed. ‘Frequent sleep apnea episodes’ was removed because it indicates an acute issue that requires investigation, if not already addressed. If it were a baseline problem that had been handled (e.g., use of oxygen or CPAP), it would already be scored elsewhere on the scale. The same reasoning applies to the removal of ‘Presents breathing difficulties that pose a risk to life.’ For the remaining two removed items, their content was already covered by other items in the same domain: ‘Seizures or altered levels of consciousness not responding to simple interventions and requiring complex interventions; drugs paired with safety protocols (e.g., oxygen administration, suctioning)’ and ‘Moderate to severe pain requiring a combination of medication (e.g., regular gabapentin and acetaminophen or morphine as needed).’ Additionally, the item ‘At high risk of skin damage, including pressure injuries, with a need to put in place preventive measures (e.g., daily monitoring of tracheostomy and gastrostomy sites, need for frequent positioning)’ was split into two items with the same scoring weight, as risks related to medical devices were overlooked. Other minor adjustments made at this stage included: slightly modifying the gradience in the *Pain*/*Irritability* domain to emphasize frequency for easier rating; adding examples to items in the *Medication Administration* domain for clarity; and removing specific reference to ‘altered level of consciousness’ in both the items and domain titled *Seizures/Altered Level of Consciousness*, as outside of episodes involving altered consciousness during seizures, regular episodes of altered consciousness should be considered acute issues needing immediate medical attention and are not common in children with complex needs.

The experts’ inter-rater reliability for the clinical vignettes rating values showed a strong agreement, with Intraclass Correlations (ICCs) of 0.91 (95% CI: 0.87–0.94) (*p* < .001) for single measures and 0.98 (95% CI: 0.97–0.98) (*p* < .001) for average measures.

### Step 3: end-user validation

The questionnaires from two end-users were rejected because they misinterpreted the task instructions and provided incomplete information. After compiling their ratings of each item’s understandability, the lowest I-CVI score was 0.8 for two items, which were slightly revised. The item ‘Clinically stable but foreseeable event(s) may jeopardize condition (e.g., high risk of sepsis)’ was changed to ‘The child is at risk for several health complications that may impair his/her condition but not necessarily cause death (e.g., high risk of aspiration pneumonia).’ Similarly, the item ‘Clinically unstable and progressing condition not responding to treatments. I would not be surprised if the child died within the next 12 months’ was revised to ‘Greater uncertainty is foreseen. I would not be surprised if the child died within the next 12 months.’ At the ACCAPED scale level, the S-CVI was 0.98. Based on the suggestions of several end-users and in agreement with all experts from the original panel, a new item was added: ‘Has a central line at home (e.g., PICC line, Port-o-Cath, Broviac, Gamcath)’ in the *Medication Administration* domain, as this is a significant factor of complexity. Almost all end users reported feeling distressed by the wording of the last domain, *Clinical Instability and/or Premature Death Risk Assessment*, which even affected their willingness to score it. It was then changed to *Clinical Instability and/or Poor Prognosis Risk Assessment*. Additionally, in this domain, end-users also found it difficult to distinguish between children with *foreseeable complications* and those with *greater uncertainty*, despite multiple reformulations of these items. Consequently, these items were rewritten to create more apparent distinctions that end users could more easily accept when scoring.

Overall, end-users exhibited slightly lower agreement with ICC values of 0.82 (95% CI: 0.68–0.93) (*p* < .001) for single measures but a strong ICC of 0.98 (95% CI: 0.95–0.99) for average measures.

### Step 4: expert panel review

No adjustments were made at this stage.

### Step 5: large-scale multisite validation and finalization

The ACCAPED scale was administered to a sample of 199 children from two tertiary hospitals in Saudi Arabia (*n* = 99) and Oman (*n* = 50), as well as a hospice in Kuwait (*n* = 50). Males made up 57.3% (*n* = 114) of the sample. The children had a mean age of 7.5 years (SD = 4.5) (Mdn = 7.5 years, IQR = 7.2). The most common conditions were neurological (*n* = 64; 32.2%), malignancy (*n* = 47; 23.6%), and genetic (*n* = 40; 19.1%) (Table 6.

**Table 6:** Children’s characteristics of the large-scale multisite validation (Saudi Arabia, Oman, Kuwait)

Sex	*n*	%
Male	114	57.3
Female	85	42.7
Diagnosis		
Malignancy	47	23.6
Non-malignancy	152	76.4
Neurological	64	32.2
Genetic	40	19.1
Respiratory	15	7.5
Hemataology	14	7.0
Hematological	13	6.5
Kidney	10	5.0
Immunological	3	1.5
Cardiovascular	2	1.0
Other^a^	6	3.0
PC Team Referral	131	65.8
Home Care Referral	19	9.6

*N* = 199. ^a^ Other: Unknown (*n* = 1, 0.5%), Prematurity (*n* = 1, 0.5%), Non-malignant brain tumor (*n* = 1, 0.5%), Juvenile dermatomyositis, Liver disease (*n* = 1, 0.5%), metabolic disease (*n* = 1, 0.5%)

Using the ACCAPED final score, categorized into three levels of PC needs complexity (Low ≤ 29, Moderate 30–55, High ≥ 56), the overall percent agreement with the intuitive *Expert Rating* was 67.3%, with a Cohen’s Kappa (k) of 0.49, indicating moderate agreement. Agreement with the corresponding categories on the PaPas scale was weaker, showing 47.2% agreement and k = 0.24, reflecting only fair concordance.

An ordinal logistic regression examined the extent to which the ACCAPED scale’s domains predicted the *Expert Rating*. The dependent variable was the *Expert Rating*, and predictors included the 11 domains of the ACCADED scale. The overall model was statistically significant, indicating that the scale’s domain scores contributed meaningfully to predicting the *Expert Rating*. Several domains emerged as strong predictors. *Mobility Status* (β = 0.49, *p* < .001), *Pain/Irritability* (β = 0.37, *p* < .001), *Clinical Instability and/or Poor Prognosis Risk Assessment* (β = 0.07, *p* < .001), and *Skin/Tissue Integrity* (β = 0.31, *p* = .008) were all positively and significantly associated with higher *Expert Ratings *(Table 7). In contrast, the other domains were not statistically significant predictors (*p* > .05). Based on these findings, adjusted scoring rules were applied to the *Mobility Status*, *Skin/Tissue integrity*, and *Pain/Irritability* domains, giving them more weight (e.g., *Mobilization*: 0 = 0, 1 = 2, 3 = 5, 5 = 10; *Pain/Irritability*: 0 = 0, 1 = 5, 2 = 10, 5 = 15). This recalibration yielded 66.3% agreement and κ = 0.46 with *Expert Ratings*—similar to the original results but with improved face validity, as it reflects clinically essential domains. Agreement with the *PaPaS scale* categories decreased further (38.2%, κ = 0.13). When optimized thresholds for complexity levels were applied to the adjusted domain scores (low ≤ 27, moderate 28–64, high ≥ 65), agreement with *Expert Ratings* improved substantially to 72.4% with K = 0.55, moving into the moderate-to-substantial range. Alignment with the PaPaS scale remained weaker (43.7% agreement, k = 0.19).

**Table 7:** Ordinal logistic regression of expert ratings based on each ACCAPED domain during the large-scale multisite validation

Predictor	β	SE	z	*p*	95% CI [LL, UL]
Breathing	0.05	0.03	1.68	0.094	-0.01, 0.10
Nutrition/Hydration	0.01	0.03	0.34	0.732	-0.05, 0.07
Seizures	-0.01	0.05	-0.31	0.754	-0.11, 0.08
Skin/Tissue integrity*	0.31	0.12	2.66	0.008	0.08, 0.53
Mobility status*	0.49	0.11	4.65	< 0.001	0.29, 0.70
Medication	-0.12	0.09	-1.29	0.197	-0.29, 0.06
Communication	-0.14	0.11	-1.27	0.204	-0.35, 0.07
Sleep/Rest	-0.04	0.12	-0.33	0.741	-0.27, 0.19
Continence/Elimination	0.01	0.05	0.19	0.850	-0.09, 0.10
Pain/Irritability*	0.37	0.10	3.71	< 0.001	0.18, 0.57
Clinical instability/Poor Prognosis Risk*	0.07	0.01	5.59	< 0.001	0.04, 0.09

*N* = 199. *β*  regression coefficient, *SE*  Standard Error, *CI*  Confidence Interval, *LL*  Lower limit, *UL*  Upper limit

*Significant predictors (*p* < .05)

Finally, an item-level agreement analysis was performed to compare how often the ACCAPED scale final scores were underestimated or overestimated relative to the *Expert Rating*, allowing for adjustments to their scoring. The scoring for the items ‘Presents dysphagia that requires interventions to ensure adequate nutrition and hydration*’* and ‘Requires a special diet to improve caloric and nutrient intake. No dysphagia present’ was lowered.

Lastly, a threshold analysis revealed that the transition between the *Moderate* and *High* complexity PC needs categories was statistically significant (cut-point = 1.33, *p* < .001). In contrast, the threshold between *Low* and *Moderate* complexity was not as substantial (cut-point = 0.44, *p* = .300), indicating that the scale more effectively distinguishes higher levels of complexity than lower ones.

No items were added or removed during this large-scale multisite validation.

## Discussion

We documented the process of translating and validating the Italian version of the ACCAPED scale into English. Experts and a sample of end-users initially evaluated its face and content validity. The tool was then tested with a large sample of children with various conditions and needs across three sites (Saudi Arabia, Oman, Kuwait). During the validation process, several adjustments were made to improve the scale’s clarity and validity. Like the original tool, the English version includes 11 domains. During the process, 16 items were removed, 3 were added, the formulations of some items were adjusted, and the cutoff thresholds were adjusted. The tool was found suitable for identifying children with PC needs and distinguishing which of them require specialized PC services. However, it was less accurate in differentiating between children with low and moderate needs than between moderate and complex needs. By involving experts and end-users from different nationalities and practice settings across countries during validation, we ensure that the scale remains relevant worldwide and is not merely ‘culturally-adapted’.

In terms of reliability, the interrater coefficients between experts and between experts and end-users remained strong throughout the validation phases. Regarding validity, factors such as mobilization, pain, instability, and skin condition were identified as significant influences on expert judgments of patient complexity compared to other domains of the tool. However, limited alignment with the *PaPaS* scale suggests that these two tools may employ different approaches to understanding patient complexity. Several items on the *PaPaS* scale appear more subjective in their ratings, which can lead to varying interpretations and scores (e.g., estimates of treatment burden and life expectancy). Additionally, this tool does not specifically assess multiple clinical needs, such as those covered by the ACCAPED scale (e.g., breathing, nutrition, seizures). These findings may explain why the complexity grading with the ACCAPED scale and the *expert ratings* tended to be higher in this study.

Identifying children with complex and palliative care needs is difficult because of their variation in age, development, and disease types. Recently, Children with Medical Complexity have been recognized as a group eligible for PC due to their complex, multiple clinical needs [24]. Their primary diagnosis alone does not fully reflect their actual complexity. The type and level of care required to manage the severity of their disease, symptoms, and related impairments give a clearer understanding of their situation. Therefore, these children are increasingly evaluated with updated diagnostic algorithms and multidimensional tools that consider not only chronic disease but also functional impairments, technology dependence, and care-coordination needs. The revised Pediatric Medical Complexity Algorithm (PMCA v3.0) identifies children with complex chronic conditions using ICD-9/ICD-10 data [25]. The Complex Chronic Conditions Classification (CCC) can also be used, often alongside PMCA; however, their overlap is moderate, highlighting that different tools may identify distinct subgroups of medically complex children [26, 27]. Recently, the PedCom Scale was developed to identify “complex chronic pediatric patients” by combining chronic-disease status with functional limitations, long-term care needs, and dependency on special treatments, thereby capturing children whose care needs are significant even if they are not flagged by diagnosis alone [28, 29]. Since studies show that different classification methods produce widely varying estimates of the prevalence of medical complexity among children, the choice of tool should be guided by the intended purpose (e.g., population health surveillance vs. individual care coordination) [26, 27].

Given the goal of assessing palliative care needs in these children, validated tools that are easy to administer and score—especially for frontline clinicians with limited specialist expertise—can support more consistent and equitable decision-making across care settings. They can also be integrated into national care frameworks. For example, implementing the ACCAPED scale in healthcare systems can help allocate resources appropriately, promote earlier referrals to specialized services such as Complex Care or PC through a clear pathway, improve access to care [30], and assist in informed clinical decision-making. The ACCAPED scale offers several advantages. It can be used in different settings and with diverse clinical populations from birth to adulthood. Compared to other tools, it emphasizes palliative care needs, which are often overlooked. It can be utilized by frontline staff with minimal training and experience. However, while it appears to be a useful screening tool, it does not fully capture the child’s and family’s PC needs. It should be part of a more comprehensive, holistic assessment that includes a clinical interview, a physical exam, and a review of the medical history. The scale focuses on clinical needs [19] and does not consider the complexity of emotional and psychosocial factors.

### Strengths and limitations

The strengths of this study include its multi-phase validation process, regular meetings and expert panel feedback with the original authors, the use of new technology (artificial intelligence) to accelerate translation, and a multisite validation phase involving a large sample of children with diverse presentations and care needs. However, limitations should be acknowledged. There may be an underrepresentation of patients with specific disease presentations. Some elements that could be included in future testing of the scale include confirmatory factor analysis and formally establishing the sensitivity and specificity of each complexity level using a larger sample. Other factors that could be used to assess the tool’s predictive validity include the number of hospitalizations per year, the number of medical services involved, mortality rates, and other existing tools used to evaluate care complexity. The scale also appears to distinguish higher levels of PC complexity more effectively than lower ones.

## Conclusion

Accurate identification of pediatric care complexity and palliative needs is essential to ensure timely and fair access to appropriate resources. The English Modified ACCAPED scale demonstrates preliminary validity and reliability, and the integration of artificial intelligence enhanced the translation process without compromising quality. The method for optimizing the scale’s psychometric properties, based on the results analysis, shows that both domain weighting and threshold adjustment were crucial for closely aligning with expert judgment, thereby reinforcing the scale’s construct validity. The tool may be beneficial for screening, but for a more comprehensive assessment of the child and family’s needs, it may not be sufficient.

Future research should include comparative studies with other assessment tools and longitudinal evaluations of clinical and service outcomes—such as quality of life, symptom burden, healthcare service utilization (e.g., emergency visits, hospitalizations, ICU admissions, and the number of medical and allied healthcare services involved, including referral to specialized PC timing)—to enhance the clarity, significance, and scoring thresholds of the items and to strengthen evidence of its clinical usefulness. Cultural variations may also be examined in the definition of ‘complex care’, which can differ across settings due to varying infrastructures, resources, and population needs.

## Supplementary Information

Supplementary Material 1.

Supplementary Material 2

## Data Availability

The datasets used and/or analysed during the current study are available from the corresponding author on reasonable request.
