# Supplementary material for: Translation and multi-site validation of the ‘Pediatric Complex Care Needs Assessment Scale’ (ACCAPED) from Italian to English
Source: BMC Palliat Care. 2026 Mar 3;25:94. doi: 10.1186/s12904-026-02031-1 (PMC13067516; doi:10.1186/s12904-026-02031-1)
Supplement: Supplementary file 1 — Supplementary Material 1. [file 12904_2026_2031_MOESM1_ESM.docx]

**Modified-Pediatric Complex Care Needs Assessment Scale (Modified-ACCAPED)**

(Translation of the ‘*Scheda di Accertamento dei Bisogni Clinico Assistenziali Complessi in Pediatria’*)

NOTE

The changes brought to the original version of the ACCAPED through the translation and validation processes can be tracked with the text in red.

| **NAME/SURNAME**  ___________________________________ | **MEDICAL RECORD NUMBER**  ___________________________________ |
| --- | --- |
| PRIMARY DISEASE  ____________________________________ | ICD-10 (OPTIONAL)  ____________________________________ |
| COMORBIDITY  ____________________________________ | ICD-10 (OPTIONAL)  ____________________________________ |
| COMORBIDITY  ____________________________________ | ICD-10 (OPTIONAL)  __________________________________ |

| **PRIMARY CAREGIVER IS:**  ☐ MOTHER  ☐ FATHER  ☐ BOTH PARENTS  ☐ OTHER FAMILY MEMBER: _________________  ☐ HEALTHCARE WORKER  ☐ OTHER _________________________________  **PRIMARY CAREGIVER SUPPORTED BY:**  ☐ THE OTHER PARENT  ☐ OTHER FAMILY MEMBER: __________________  ☐ HEALTHCARE WORKER  ☐ OTHER _________________________________ | **EQUIPMENT/DEVICE(S):**  ☐ AIDS FOR MOBILIZATION _______________  ☐ DEVICE FOR MOTOR FUNCTION  ____________________________________  ☐ DEVICE FOR RESPIRATORY FUNCTION  ☐ NASAL CANNULA: _____ LITER/MIN  ☐ FACE MASK: ______ LITER/MIN OR %  ☐ HIGH FLOW: ______ LITER/MIN OR %  ☐ CPAP ☐ BIPAP  ☐ MECHANICAL VENTILATOR  ☐ MONITOR/OXIMETER  ☐ FEEDING PUMP  ☐ SUCTION MACHINE  ☐ OTHER ______________________________ |
| --- | --- |
| **CHILD’S LEGAL DECISION-MAKER:**  ☐ MOTHER  ☐ FATHER  ☐ BOTH PARENTS  ☐ OTHER FAMILY MEMBER: _________________  ☐ HEALTHCARE WORKER  ☐ OTHER _________________________________ | **IN SITU MEDICAL DEVICE(S):**  ☐ TRACHEOSTOMY  ☐ NASOGASTRIC TUBE  ☐ GASTROSTOMY ☐ JEJUNOSTOMY  ☐ CENTRAL VENOUS CATHETER  ☐ PICC LINE/MIDLINE  ☐ PORT-O-CATH  ☐ GAMCATH  ☐ BROVIAC  ☐ OTHER ______________________________ |

DATE (DD/MM/YYYY): _____ / _____ / _________ SIGNATURE: __________________________________________

**Pediatrics Complex Care Needs Assessment Scale (ACCAPED)**

(Translation of the ‘*Scheda di Accertamento dei Bisogni Clinico Assistenziali Complessi in Pediatria’*)

**INTRODUCTION**

The Pediatric Complex Care Needs Assessment Scale (ACCAPED) is a rapid triage tool that was created to identify the needs of children diagnosed with incurable and/or chronic conditions associated with complex clinical care.

The main aims of the ACCA PED scale are:

• To assess the complexity of the child's clinical needs;

• To identify, based on these needs, the appropriate Palliative Pediatric Care (PPC) expertise required;

~~• To propose a standardized and easy-to-use tool for healthcare providers to identify PPC needs.~~

**The use of the ACCA PED scale doesn’t exclude the necessity of carrying a comprehensive assessment of the child’s needs. The child and caregivers’ psychosocial, spiritual, ethical, economic, and social needs must be considered.**

**SCORING**

The ACCAPED scale consists of (10) clinical needs categories (breathing, nutrition, seizures and altered level of consciousness, mobility, communication, sleep and rest, continence and elimination, medication administration, pain, clinical instability and risks of premature death) that are scored individually according to their level of complexity.

After, the scores of each of the (10) clinical needs categories are summed up to provide a final score. With this score, the child is categorized as having either *low*, *medium*, or *high* complexity clinical needs.

**INTERPRETATION**

| **Type of Patients** | **Global Score** | **Type of PPC Service Required** |
| --- | --- | --- |
| **Low** complexity | ≤ ~~29~~ 27 | Level 1: Primary Palliative Care  Primary care, community care, local healthcare facilities, Family Medicine, General Pediatrics  *Provided by first-line healthcare providers* |
| **Moderate** complexity | ~~30-49~~ ~~55~~ 28-64 | Level 2: General Complex/Palliative Care  Secondary care, Hospital, medical specialties  *Provided by healthcare with additional training in complex/palliative care* |
| **High** complexity | ≥ ~~50~~ ~~55~~ 64 | Level 3: Specialized Palliative Care  Tertiary care, hospitals, Hospice, Palliative care home  *Provided by Complex/Palliative Care specialists* |

**BREATHING ASSESSMENT**

| **ORIGINAL**  (It.) | **FORWARD**  (Eng.) | **BACKWARD**  (It.) | **BACKWARD REVIEW** (Eng.) | **EXPERT PANEL #1** | **EXPERT PANEL #2** | **EXPERT PANEL #3** | **END USERS** |
| --- | --- | --- | --- | --- | --- | --- | --- |
| Respiro normale (appropriato all'età), ~~nessun episodio di alterazione del respiro.~~ Uso routinario di inalatori, nebulizzatori ecc. | Normal breathing (parameters appropriate for age). May use inhalers or nebulizers. | Respirazione normale (parametri appropriati per l'età). Può utilizzare inhalatori o nebulizzatori. | Normal breathing (parameters appropriate for age). May use inhalers or nebulizers. | Normal breathing. ~~(parameters appropriate for age).~~ May use inhalers or nebulizers. | Normal breathing. May use inhalers or nebulizers. | Normal breathing. May use inhalers or nebulizers. | Normal breathing. May use inhalers or nebulizers. |
| Episodi di broncospasmo e tosse o difficoltà respiratorie, che non rispondono a trattamenti autogestiti. | Episodes of bronchospasm and cough or breathing difficulties, not respond to self-administered treatments. | Episodi di broncospasmo e tosse o difficoltà respiratorie non rispondenti a trattamenti auto-somministrati. | Episodes of bronchospasm and cough or breathing difficulties, not respond to self-administered treatments. | Episodes of bronchospasm/cough or breathing difficulties, not responding to self-administered treatments. | Episodes of bronchospasm/cough or breathing difficulties, not responding to self-administered treatments. | Episodes of bronchospasm/cough or breathing difficulties, not responding to self-administered treatments. | Episodes of bronchospasm/cough or breathing difficulties, not responding to self-administered treatments. |
| Necessita di ossigenoterapia | Requires oxygen therapy (either by nasal cannula, face mask, or non-rebreather mask; continuously or as needed). | Richiede terapia con ossigeno (tramite cannula nasale, maschera facciale o maschera non-rigenerante; continuamente o secondo necessità). | Requires oxygen therapy (either by nasal cannula, face mask, or non-rebreather mask; continuously or as needed). | Requires oxygen therapy (~~either by~~ by nasal cannula or face mask; ~~non-rebreather mask~~ continuously or as needed). | Requires oxygen therapy (nasal cannula or face mask; continuously or as needed). | Requires oxygen therapy (nasal cannula or face mask; continuously or as needed). | Uses a nasal cannula (NC) or a face mask (FM) (continuously or as needed). |
| Maggiore suscettibilità alle infezioni polmonari. | Increased susceptibility to lung infections. | Aumentata suscettibilità alle infezioni polmonari. | Increased susceptibility to lung infections. | ~~Increased susceptibility to lung infections.~~ |  |  |  |
| Necessita di fisioterapia respiratoria. | Requires chest physiotherapy. | Richiede fisioterapia toracica. | Requires chest physiotherapy. | Requires regular chest physiotherapy. | Requires regular chest physiotherapy. | Requires regular chest physiotherapy. | Requires regular chest physiotherapy. |
| Portatore di tracheostomia con respiro autonomo. | Breathes independently through a tracheostomy. | Respira autonomamente attraverso una tracheostomia. | Breathes independently through a tracheostomy. | Breathes independently through a tracheostomy. | Breathes independently through a tracheostomy. | Breathes independently through a tracheostomy. | Breathes independently through a tracheostomy (with or without oxygen therapy) |
| Presenta anomalia delle vie aeree con respiro autonomo. | Present an airway abnormality but can breathe independently. | Presenta un'anomalia delle vie respiratorie ma può respirare autonomamente. | Present an airway abnormality but can breathe independently. | ~~Present an airway abnormality but can breathe independently~~ |  |  |  |
| Respirazione autonoma valida durante il giorno, durante la notte utilizzo non salvavita di supporto ventilatorio (il paziente non risente della eventuale sospensione della ventilazione, anche fino a 48h). | Can breathe independently during daytime, but requires non-lifesaving ventilation support at nighttime (e.g. CPAP), it could be interrupted for up to 48 hours without foreseeable consequences. | Può respirare autonomamente durante il giorno, ma richiede supporto ventilatorio non salvavita di notte (ad esempio, CPAP), che potrebbe essere interrotto fino a 48 ore senza conseguenze prevedibili. | Can breathe independently during daytime, but requires non-lifesaving ventilation support at nighttime (e.g. CPAP), it could be interrupted for up to 48 hours without foreseeable consequences. | Can breathe independently during daytime, but requires non-lifesaving ventilation support at nighttime (e.g. CPAP). It could be interrupted for up to 48 hours without foreseeable serious consequences. | Can breathe independently during daytime, but requires non-lifesaving ventilation support at nighttime (e.g. CPAP). It could be interrupted for up to 48 hours without foreseeable serious consequences. | Can breathe independently during daytime, but requires non-lifesaving ventilation support at nighttime (e.g. CPAP). It could be interrupted for up to 48 hours without foreseeable serious consequences. | Can breathe independently during daytime, but requires ~~non-lifesaving~~ ventilation support at nighttime (CPAP). It could be interrupted for up to 48 hours without f~~oreseeable~~ serious consequences. |
|  |  |  |  | Uses a non-rebreather mask. | Uses a non-rebreather mask. | Uses a non-rebreather mask. | Uses a non-rebreather mask (NRM). |
| Ossigenoterapia ad Alti flussi. | Uses High Flow Nasal Cannula (HFNC) oxygen therapy. | Utilizza terapia con cannula nasale ad alto flusso (HFNC) di ossigeno. | Uses High Flow Nasal Cannula (HFNC) oxygen therapy. | Uses High Flow Nasal Cannula (HFNC) ~~oxygen therapy~~. | Uses High Flow Nasal Cannula (HFNC). | Uses High Flow Nasal Cannula (HFNC). | Uses a High Flow Nasal Cannula (HFNC). |
| Apnee frequenti. | Frequent apnea episodes. | Episodi frequenti di apnea. | Frequent apnea episodes. | Frequent apnea episodes. | Frequent sleep apnea episodes. | ~~Frequent sleep apnea episodes.~~ |  |
| Difficoltà respiratoria che comporta pericolo di vita. | Presents breathing difficulties that pose a risk to life. | Presenta difficoltà respiratorie che comportano un rischio per la vita. | Presents breathing difficulties that pose a risk to life. | Presents breathing difficulties that pose a risk to life. | Presents breathing difficulties that pose a risk to life. | ~~Presents breathing difficulties that pose a risk to life.~~ |  |
| Portatore di tracheostomia che richiede frequenti aspirazioni. | Has a tracheostomy that requires frequent suctioning. | Ha una tracheostomia che richiede frequenti aspirazioni. | Has a tracheostomy that requires frequent suctioning. | ~~Has a tracheostomy that requires frequent suctioning.~~ |  |  |  |
| Ventilazione notturna salvavita (potrebbe sopravvivere alla disconnessione accidentale ma con sintomatologia che richiederebbe il supporto ospedaliero). | Requires nighttime life-saving ventilation (BiPAP). Could survive an accidental disconnection (e.g., between 1 to 3 hours), but this would probably cause a deterioration requiring hospital support). | Richiede ventilazione salvavita notturna (BiPAP). Potrebbe sopravvivere a una disconnessione accidentale (ad esempio, tra 1 e 3 ore), ma ciò potrebbe causare probabilmente un deterioramento che richiede il supporto ospedaliero. | Requires nighttime life-saving ventilation (BiPAP). Could survive an accidental disconnection (e.g., between 1 to 3 hours), but this would probably cause a deterioration requiring hospital support). | Requires nighttime life-saving ventilation (BiPAP). Could survive an accidental disconnection (e.g., between 1 to 3 hours), but this would probably cause a deterioration requiring hospital support). | Requires nighttime life-saving ventilation (BiPAP). Could survive an accidental disconnection (e.g., between 1 to 3 hours), but this would probably cause a deterioration requiring hospital support). | Requires nighttime life-saving ventilation (BiPAP). Could survive an accidental disconnection (e.g., between 1 to 3 hours), but this would probably cause a deterioration requiring hospital support). | Requires nighttime ~~life-saving~~ ventilation (BiPAP). Could survive an accidental disconnection (e.g. 1 to 3 hour(s), but this would cause a deterioration requiring hospital support). |
| Abbondanti secrezioni che richiedono frequenti aspirazioni. | Presents abundant secretions that require frequent suctioning. | Presenta abbondanti secrezioni che richiedono frequenti aspirazioni. | Presents abundant secretions that require frequent suctioning. | Presents abundant secretions that require frequent suctioning (with or without a tracheostomy). | Presents abundant secretions that require frequent suctioning (with or without a tracheostomy). | Presents abundant secretions that require frequent suctioning (with or without a tracheostomy). | Presents abundant secretions that require frequent suctioning (with or without a tracheostomy). |
| Mancanza di drive respiratorio quando dorme o in fase di incoscienza e richiede ventilazione salvavita mentre dorme. | Does not have a respiratory drive when asleep or unconscious and requires a BiPAP support in these circumstances. A disconnection could be fatal. | Non ha stimolo respiratorio durante il sonno o in stato di incoscienza e richiede supporto BiPAP in tali circostanze. Una disconnessione potrebbe essere fatale. | Does not have a respiratory drive when asleep or unconscious and requires a BiPAP support in these circumstances. A disconnection could be fatal. | Does not have a respiratory drive when asleep or unconscious and requires a BiPAP support in these circumstances. A disconnection could be fatal. | Does not have a respiratory drive when asleep or unconscious and requires a BiPAP support in these circumstances. A disconnection could be fatal. | Does not have a respiratory drive when asleep or unconscious and requires a BiPAP support in these circumstances. A disconnection could be fatal. | Does not have a respiratory drive when asleep or unconscious and requires a BiPAP support in these circumstances. A disconnection could be fatal. |
| Incapace di respiro autonomo, richiede ventilazione meccanica continuativa | Requires continuous mechanical ventilation due to an inability to breathe independently. | Richiede ventilazione meccanica continua a causa dell'incapacità di respirare autonomamente. | Requires continuous mechanical ventilation due to an inability to breathe independently. | Requires ~~continuous~~ mechanical ventilation due to an inability to breathe independently. | Requires ~~continuous~~ mechanical ventilation due to an inability to breathe independently. | Requires mechanical ventilation due to an inability to breathe independently. | Requires mechanical ventilation. ~~due to an inability to breathe independently.~~ |
| Tracheostomia altamente instabile, frequenti occlusioni, difficoltà alla sostituzione della cannula. | Unstable tracheostomy, frequent occlusions, and difficulties in replacing the cannula. | Tracheostomia instabile, frequenti occlusioni e difficoltà nella sostituzione della cannula. | Unstable tracheostomy, frequent occlusions, and difficulties in replacing the cannula. | Unstable tracheostomy, frequent occlusions, and difficulties in replacing the inner cannula. | Unstable tracheostomy, frequent occlusions, and difficulties in replacing the inner cannula. | Unstable tracheostomy, frequent occlusions, and difficulties in replacing the inner cannula. | Unstable tracheostomy, frequent occlusions, and difficulties in replacing the inner cannula. |

**NUTRITIONAL ASSESSMENT**

| **ORIGINAL** | **FORWARD** | **BACKWARD** | **RECONCILIATION** | **EXPERT PANEL #1** | **EXPERT PANEL #2** | **EXPERT PANEL #3** | **END-USERS** |
| --- | --- | --- | --- | --- | --- | --- | --- |
| Autonomo nell'assunzione di cibo e bevande per via orale, adeguata all'età. | Capacity to feed orally is adequate for their age (food/fluids). | La capacità di alimentarsi per via orale è adeguata per l'età (cibo/liquidi). | Capacity to feed orally is adequate for their age (food/fluids). | Capacity to feed orally is adequate for age (food/fluids). | Capacity to feed orally is adequate for age (food/fluids). | Capacity to feed orally is adequate for age (food/fluids). | Capacity to feed orally is adequate for age (food/fluids). |
| ~~Necessita di assistenza maggiore rispetto a quella prevista per la sua età~~ O necessita di supervisione, consigli e incoraggiamento, per l'assunzione di cibo e bevande, maggiore rispetto a quella prevista per la sua età. | Requires supervision, instructions, and encouragement to feed orally (food/fluid), more than expected for their age. | Richiede supervisione, istruzioni e incoraggiamento per l'alimentazione orale (cibo/liquidi), più del previsto per l'età. | Requires supervision, instructions, and encouragement to feed orally (food/fluid), more than expected for their age. | Requires supervision, instructions, and encouragement to feed orally (food/fluid), more than expected for age. | Requires supervision, instructions, and encouragement to feed orally (food/fluid), more than expected for age. | Requires supervision, instructions, and encouragement to feed orally (food/fluid), more than expected for age. | Requires supervision, instructions, and encouragement to feed orally (food/fluid), more than expected for age. |
| Necessita di assistenza completa nell'alimentazione per os, con durata dei pasti anche molto prolungata. | Requires complete assistance to feed orally, with mealtimes taking a very long time. | Richiede assistenza completa per l'alimentazione orale, con pasti che richiedono molto tempo. | Requires complete assistance to feed orally, with mealtimes taking a very long time. | Requires complete assistance to feed orally, with mealtimes taking a very long time (inappropriate for age). | Requires complete assistance to feed orally, with mealtimes taking a very long time (inappropriate for age). | Requires complete assistance to feed orally, with mealtimes taking a very long time (inappropriate for age). | Requires complete assistance to feed orally, with mealtimes taking a very long time (inappropriate for age). |
| Dieta speciale. | Requires a special diet (e.g., to improve caloric and nutrient intake). | Richiede una dieta speciale (ad esempio, per migliorare l'apporto calorico e nutrizionale). | Requires a special diet (e.g., to improve caloric and nutrient intake). | Requires a special diet to improve *caloric* and *nutrient* intake (e.g. hypercaloric, low sodium, ketogenic diet). No dysphagia present. | Requires a special diet to improve *caloric* and *nutrient* intake (e.g. hypercaloric, low sodium, ketogenic diet). No dysphagia present. | Requires a special diet to improve *caloric* and *nutrient* intake (e.g. hypercaloric, low sodium, ketogenic diet). No dysphagia present. | Requires a special diet to improve *caloric* and *nutrient* intake (e.g. hypercaloric, low sodium, ketogenic diet). ~~No dysphagia present.~~ |
| Incapace di assumere cibi solidi o liquidi per via orale (portatore di gastrostomia, digiunostomia, sondino naso gastrico). | Unable to feed orally (food/fluid). An artificial feeding device is used (e.g., gastrostomy, jejunostomy, nasogastric tube). | Incapace di alimentarsi per via orale (cibo/liquidi). Viene utilizzato un dispositivo di alimentazione artificiale (ad esempio, gastrostomia, jejunostomia, tubo nasogastrico). | Unable to feed orally (food/fluid). An artificial feeding device is used (e.g., gastrostomy, jejunostomy, nasogastric tube). | Unable to feed orally (food/fluid). An artificial tube feeding is required (e.g. gastrostomy, jejunostomy, nasogastric tube). | Unable to feed orally (food/fluid). An artificial tube feeding is required (e.g. gastrostomy, jejunostomy, or nasogastric tube). | Unable to feed orally (food/fluid). An artificial tube feeding is required (e.g. gastrostomy, jejunostomy, nasogastric tube). | ~~Unable to feed orally (food/fluid).~~ Uses a tube feeding for all or a portion of the nutrition (e.g. gastrostomy, jejunostomy, or nasogastric tube). |
| Disfagia che richiede interventi aggiuntivi per garantire un’adeguata nutrizione o idratazione. | Dysphagia that requires interventions to ensure adequate nutrition and hydration. | Disfagia che richiede interventi per garantire un'adeguata nutrizione e idratazione. | Dysphagia that requires interventions to ensure adequate nutrition and hydration. | Dysphagia that requires interventions to ensure adequate nutrition and hydration. | Dysphagia that requires interventions to ensure adequate nutrition and hydration (e.g. soft diet or puree diet, thickened fluids). | Dysphagia that requires interventions to ensure adequate nutrition and hydration (e.g. soft diet or puree diet, thickened fluids). | Presents dysphagia requiring interventions to ensure adequate nutrition and hydration (e.g. soft or puree diet, thickened fluids). |
| Trend della curva ponderale in calo tale da richiedere interventi atti a garantire un’adeguata nutrizione o idratazione. | Unexpected weight loss that requires interventions to ensure adequate nutrition and hydration. | Perdita di peso inaspettata che richiede interventi per garantire un'adeguata nutrizione e idratazione. | Unexpected weight loss that requires interventions to ensure adequate nutrition and hydration. | Unexpected weight loss that requires interventions to ensure adequate nutrition and hydration. | ~~Unexpected weight loss that requires interventions to ensure adequate nutrition and hydration.~~ |  |  |
| Idratazione e/o alimentazione totalmente per via endovenosa. | Hydration and/or nutrition administered entirely intravenously. | Idratazione e/o nutrizione somministrate interamente per via endovenosa. | Hydration and/or nutrition administered entirely intravenously. | Intravenous hydration and/or Total Parenteral Nutrition (TPN). | Intravenous hydration and/or Total Parenteral Nutrition (TPN). | Intravenous hydration and/or Total Parenteral Nutrition (TPN). | Intravenous hydration (IV) and/or Total Parenteral Nutrition (TPN). |

**SEIZURES ~~AND ALTERED STATE OF CONSCIOUSNESS~~ ASSESSMENT**

| **ORIGINAL** | **FORWARD** | **BACKWARD** | **RECONCILIATION** | **EXPERT PANEL #1** | **EXPERT PANEL #2** | **EXPERT PANEL #3** | **END-USERS** |
| --- | --- | --- | --- | --- | --- | --- | --- |
| Nessuna presenza di convulsioni o alterazione dello stato di coscienza. | Absence of seizures or altered level of consciousness (not on anticonvulsant therapy). | Assenza di crisi convulsive o alterazione del livello di coscienza (senza terapia anticonvulsivante). | Absence of seizures or altered level of consciousness (not on anticonvulsant therapy). | Absence of seizures or altered level of consciousness (not on anticonvulsant therapy). | Absence of seizures or altered level of consciousness (not on anticonvulsant therapy). | Absence of seizures ~~or altered level of consciousness~~ (not on anticonvulsant therapy). | No history of seizures. |
| Crisi convulsive ben controllate con la terapia anticomiziale. | Absence of seizures on anticonvulsant therapy. | Assenza di crisi convulsive sotto terapia anticonvulsivante. | Absence of seizures (on anticonvulsant therapy). | Absence of seizures due to current anticonvulsant therapy. | Absence of seizures or altered level of consciousness (not on anticonvulsant therapy). | Absence of seizures due to current anticonvulsant therapy. | Absence of seizures (on anticonvulsant therapy). |
| Crisi convulsive o alterazioni dello stato di coscienza occasionali negli ultimi tre mesi (con o senza terapia anticomiziale). | Occasional seizures or altered level of consciousness in the last 3 months (with or without anticonvulsant treatment). | Crisi occasionali o alterazione del livello di coscienza negli ultimi 3 mesi (con o senza trattamento anticonvulsivante). | Occasional seizures or altered level of consciousness in the last 3 months (with or without anticonvulsant treatment). | *Occasional* seizures or altered level of consciousness (up to a few times per month) that respond well to simple interventions by family caregivers or healthcare providers. | *Occasional* seizures or altered level of consciousness (up to a few times per month) that respond well to simple interventions by family caregivers or healthcare providers. | *Occasional* seizures ~~or altered level of consciousness~~ (up to a few times per month) that respond well to simple interventions by family caregivers or healthcare providers. | *Occasional* seizures (up to a few times per month) that respond well to simple interventions by family caregivers or healthcare providers. |
| Crisi convulsive O alterazioni dello stato di coscienza che richiedono frequentemente interventi specifici con buona risposta alla terapia (più di una volta al mese). | Regular seizures or altered level of consciousness up to a few times per month that respond well to therapy. | Crisi regolari o alterazione del livello di coscienza fino a qualche volta al mese che rispondono bene alla terapia. | Regular seizures or altered level of consciousness up to a few times per month that respond well to therapy. | ~~Regular seizures or altered level of consciousness up to a few times per month that respond well to therapy.~~ |  |  |  |
| Crisi convulsive o alterazione dello stato di coscienza frequenti (anche ogni giorno ) che si risolvono con l'intervento da parte di personale/care giver competente. | Frequent seizures or altered level of consciousness (up to daily) that respond well to therapy. | Crisi frequenti o alterazione del livello di coscienza (fino a quotidiane) che rispondono bene alla terapia. | Frequent seizures or altered level of consciousness (up to daily) that respond well to caregivers or healthcare providers interventions. | *Frequent* seizures or altered level of consciousness (up to daily) that respond well to interventions by family caregivers or healthcare providers. | *Frequent* seizures or altered level of consciousness (up to daily) that respond well to interventions by family caregivers or healthcare providers. | *Frequent* seizures ~~or altered level of consciousness~~ (up to daily) that respond well to interventions by family caregivers or healthcare providers. | *Frequent* seizures (up to daily) that respond well to interventions by family caregivers or healthcare providers. |
| Crisi convulsive o alterazione dello stato di coscienza frequenti (più di 4 volte a settimana), che richiedono interventi complessi (somministrazione di farmaci, protocolli di sicurezza es. somministrazione di ossigeno e aspirazione). | Frequent seizures or altered levels of consciousness (more than 4 times a week) requiring complex interventions: drugs paired with safety protocols (e.g., oxygen administration, suctioning). | Crisi frequenti o alterazione del livello di coscienza (più di 4 volte a settimana) che richiedono interventi complessi: farmaci associati a protocolli di sicurezza (ad esempio, somministrazione di ossigeno, aspirazione). | Frequent seizures or altered levels of consciousness (more than 4 times a week) requiring complex interventions: drugs paired with safety protocols (e.g., oxygen administration, suctioning). | ~~Frequent~~ Seizures or altered levels of consciousness ~~(more than 4 times a week)~~ not responding to simple interventions and requiring complex interventions: drugs paired with safety protocols (e.g. oxygen administration, suctioning). | Seizures or altered levels of consciousness not responding to simple interventions and requiring complex interventions: drugs paired with safety protocols (e.g. oxygen administration, suctioning). | ~~Seizures or altered levels of consciousness not responding to simple interventions and requiring complex interventions: drugs paired with safety protocols (e.g. oxygen administration, suctioning).~~ |  |
| Il bambino in questa categoria è instabile. | The child is at home and unstable due to uncontrollable seizures. | Il bambino è a casa ed è instabile a causa di crisi incontrollabili. | The child is unstable due to uncontrollable seizures. | ~~The child is unstable due to uncontrollable seizures.~~ |  |  |  |
| Crisi convulsive o alterazione dello stato di coscienza frequenti (anche ogni giorno ) difficilmente controllabili che comportano un alto rischio per la vita (ad esempio, resistente alla somministrazione di Midazolam/Buccolam). | Persistent seizures or altered level of consciousness (up to daily) refractory to therapy and posing a high risk to life (e.g., resistance to Midazolam, lorazepam, diazepam). | Crisi persistenti o alterazione del livello di coscienza (fino a quotidiane) refrattarie alla terapia e che comportano un elevato rischio per la vita (ad esempio, resistenza a Midazolam, lorazepam, diazepam). | Persistent seizures or altered level of consciousness (up to daily) refractory to therapy and posing a high risk to life (e.g., resistance to Midazolam, lorazepam, diazepam). | Persistent seizures or altered level of consciousness (up to daily) refractory to therapy and posing a high risk to life (e.g. resistance to midazolam, lorazepam, diazepam). | Persistent seizures or altered level of consciousness (up to daily) refractory to therapy and posing a high risk to life (e.g. resistance to midazolam, lorazepam, diazepam). | Uncontrolled seizures ~~and altered level of consciousness~~ (up to daily) refractory to therapy and requiring additional safety protocols (e.g. oxygen administration, suctioning) | Uncontrolled seizures ~~(up to daily) refractory to therapy and~~ requiring ~~additional~~ complex muti-drug regime and additional interventions ~~safety protocols~~ (e.g. oxygen, ~~administration~~ suctioning) |

**SKIN AND TISSUE INTEGRITY ASSESSMENT**

| **ORIGINAL** | **FORWARD** | **BACKWARD** | **RECONCILIATION** | **EXPERT PANEL #1** | **EXPERT PANEL #2** | **EXPERT PANEL #3** | **END-USERS** |
| --- | --- | --- | --- | --- | --- | --- | --- |
| Nessun segno di lesioni da pressione o compromissione della cute. | No signs of pressure injuries nor compromised skin integrity. | Nessun segno di lesioni da pressione o compromissione dell'integrità cutanea. | No signs of pressure injuries nor compromised skin integrity. | No signs of pressure injuries nor compromised skin integrity. | No signs of pressure injuries nor compromised skin integrity. | No signs of pressure injuries nor compromised skin integrity. | No signs of pressure injuries nor compromised skin integrity. |
| Presenta iperemia della cute da pressione o lesioni minori. | Skin redness from a pressure injury or minor skin lesions. | Rossore cutaneo da lesioni da pressione o lesioni cutanee minori. | Skin redness from a pressure injury or minor skin lesions. | Skin redness from a pressure injury or presence of minor skin lesions. | Skin redness from a pressure injury or presence of minor skin lesions. | Skin redness from a pressure injury or presence of minor skin lesions. | Skin redness from a pressure injury or presence of minor skin lesions. |
| Compromissione della cute che richiede un controllo clinico almeno di una volta a settimana (~~es. mascherina della NIV).~~ | Compromised skin integrity, requires at least weekly clinical monitoring. | Integrità cutanea compromessa, richiede monitoraggio clinico almeno settimanale. | Compromised skin integrity, requires at least weekly clinical monitoring. | ~~Compromised skin integrity, requires at least weekly clinical monitoring.~~ |  |  |  |
| Ferite aperte, che rispondono al trattamento. | Open wound(s), responding to treatment. | Ferita(e) aperta(e), che rispondono al trattamento. | Open wound(s), responding to treatment. | Open wound(s) or compromised skin integrity, responding to first-line treatment (e.g. simple dressing). | Open wound(s) or compromised skin integrity, responding to first-line treatment (e.g. simple dressing). | Open wound(s) or compromised skin integrity, responding to first-line treatment (e.g. simple dressing). | Open wound(s) or compromised skin integrity, responding to first-line treatment (e.g. simple dressing). |
| Compromissione della cute, che richiede un controllo giornaliero e che risponde ai trattamenti. | Compromised skin integrity, requires daily monitoring and responding to treatment. | Integrità cutanea compromessa, richiede monitoraggio giornaliero e trattamento. | Compromised skin integrity, requires daily monitoring and responding to treatment. |  |  |  |  |
| Alto rischio di danno alla cute, che richiede interventi preventivi, volti a evitare la compromissione dell'integrità cutanea (es. controllo quotidiano dello stoma della trachestomia o gastrostomia). | At high risk of skin damage, including pressure injuries, with a need to put in place preventive measures (e.g., daily monitoring of tracheostomy/gastrostomy site, regular re-positioning). | Alto rischio di danni cutanei, inclusi le lesioni da pressione, con necessità di adottare misure preventive (ad esempio, monitoraggio giornaliero del sito della tracheostomia/gastrostomia, riposizionamento regolare). | At high risk of skin damage, including pressure injuries, with a need to put in place preventive measures (e.g., daily monitoring of tracheostomy/gastrostomy site, regular re-positioning). | At high risk of skin damage, including pressure injuries, with a need to put in place preventive measures (e.g. daily monitoring of tracheostomy/gastrostomy site, need for frequent positioning). | At high risk of skin damage, including pressure injuries, with a need to put in place preventive measures (e.g. daily monitoring of tracheostomy/gastrostomy site, need for frequent positioning). | At high risk of pressure injuries due to immobility, requiring preventive measures (e.g. regular re-positioning, special mattress). | At high risk of pressure injuries due to immobility, requiring preventive measures (e.g. ~~regular re-~~positioning, special mattress). |
|  |  |  |  |  |  | At high risk for medical device-related pressure injuries requiring frequent monitoring (e.g. gastrostomy site, BiPAP/CPAP, oxygen cannula, NGT). | At high risk for medical device-related pressure injuries requiring frequent monitoring (e.g. gastrostomy site, BiPAP/CPAP, oxygen cannula, naso-gastric tube, central line). |
| Ferite aperte che non rispondono ai trattamenti. | Open wound(s), not responding to treatment. | Ferita(e) aperta(e), non rispondenti al trattamento. | Open wound(s), not responding to treatment. | Open wound(s) or compromised skin integrity not responding to first-line treatment, requiring specialized treatment (e.g. specialized dressing, wound care specialists). | Open wound(s) or compromised skin integrity not responding to first-line treatment, requiring specialized treatment (e.g. specialized dressing, wound care specialists). | Open wound(s), burns or compromised skin integrity not responding to first-line treatment, requiring specialized treatment (e.g. specialized dressing, wound care specialists, pain management). | Open wound(s), burns or compromised skin integrity not responding to first-line treatment, requiring specialized treatment (e.g. specialized dressing, wound care specialists, pain management). |
| Compromissione della cute che richiede almeno un controllo giornaliero e trattamenti specifici es. stomia arrossata | Compromised skin integrity, requires specific treatments (e.g. skin irritation around a stoma) and at least daily clinical monitoring. | Integrità cutanea compromessa, richiede trattamenti specifici (ad esempio, irritazione cutanea intorno a una stomia) e monitoraggio clinico almeno quotidiano. | Compromised skin integrity, requires specific treatments (e.g. skin irritation around a stoma) and at least daily clinical monitoring. |  |  |  |  |
| Medicazioni specifiche programmate più volte alla settimana, con risposta ai trattamenti ~~es. granuloma.~~ | Wound(s) requiring specialized dressing(s) multiple times per week, respond to treatment. | Ferita(e) che richiedono applicazione di bende specializzate più volte a settimana, rispondono al trattamento. | Wound(s) requiring specialized dressing(s) multiple times per week, respond to treatment. | ~~Wound(s) requiring specialized dressing(s) multiple times per week, respond to treatment.~~ |  |  |  |
| ~~Compromissione della cute o ustioni,~~ che richiedono interventi complessi e dolorosi per un periodo prolungato. | Wound(s) or burns that require complex and painful interventions for a prolonged period. | Ferita(e) o ustioni che richiedono interventi complessi e dolorosi per un periodo prolungato. | Wound(s) or burns that require complex and painful interventions for a prolonged period. | Wound(s) or burns that require complex/painful interventions for a prolonged period. | Wound(s) or burns that require complex/painful interventions for a prolonged period. | ~~Wound(s) or burns that require complex/painful interventions for a prolonged period.~~ |  |

**MEDICATION ADMINISTRATION ASSESSMENT**

| **ORIGINAL** | **FORWARD** | **BACKWARD** | **RECONCILIATION** | **EXPERT PANEL #1** | **EXPERT PANEL #2** | **EXPERT PANEL #3** | **END USERS** |
| --- | --- | --- | --- | --- | --- | --- | --- |
| Non necessita di somministrazione di farmaci per la patologia. | Not requiring medication for their medical condition(s). | Non richiede farmaci per le sue condizioni mediche. | Not requiring medication for their medical condition(s). | Not requiring medication for their baseline medical condition(s). | Not requiring medication for their baseline medical condition(s). | Not requiring medication for their baseline medical condition(s). | Not requiring medication for their baseline medical condition(s). |
| Somministrazione di farmaci che richiede una persona adeguatamente addestrata. | Administration of medication requires a trained caregiver. | L'amministrazione di farmaci richiede un caregiver addestrato. | Administration of medication requires a trained caregiver. | Requires medication for their baseline medical condition(s). Can easily be administered by a trained family caregiver. | Requires medication for their baseline medical condition(s). Can easily be administered by a trained family caregiver. | Requires medication for their baseline medical condition(s). Can be taken by the child with supervision or easily administered by a trained family caregiver. | Requires medication for their baseline medical condition(s). Can be taken by the child with supervision or easily administered by a trained family caregiver. |
| Somministrazione di farmaci che può richiedere, causa di fluttuazione della condizione clinica la supervisione da parte di personale sanitario. | Due to a fluctuating clinical condition, medication administration requires healthcare provider's supervision. | A causa di una condizione clinica fluttuante, l'amministrazione di farmaci richiede la supervisione di un operatore sanitario. | Due to a fluctuating clinical condition, medication administration requires healthcare provider's supervision. | Requires medication for their baseline medical condition(s). Can be administered by a trained family caregiver but due to a fluctuating condition requires frequent support from a healthcare provider (e.g. frequent medical visits). | Requires medication for their baseline medical condition(s). Can be administered by a trained family caregiver but due to a fluctuating condition requires frequent support from a healthcare provider (e.g. frequent medical visits). | Requires medication for their baseline medical condition(s). Some medication needs to be administered by a healthcare provider (e.g. home care support, clinic, day unit). | Requires medication for their baseline medical condition(s). Some medication needs to be administered by a healthcare provider in an Outpatient setting (e.g. home care support, clinic, day unit). |
| Somministrazione di farmaci che richiedono interventi quotidiani da parte di una persona competente ed il riferimento ad un medico per garantire una gestione efficace dei sintomi associati ad una condizione rapidamente modificabile/deteriorabile. | Due to a rapidly fluctuating/progressing condition, in addition to a daily medication administration by a trained caregiver, a frequent medical follow-up is required to achieve effective symptom management. | A causa di una condizione clinica che si evolve rapidamente, oltre all'amministrazione quotidiana dei farmaci da parte di un caregiver addestrato, è necessario un frequente follow-up medico per ottenere una gestione efficace dei sintomi. | Due to a rapidly fluctuating/progressing condition, in addition to a daily medication administration by a trained caregiver, a frequent medical follow-up is required to achieve effective symptom management. | Requires medication for their baseline medical condition(s). Needs to be administered in a healthcare setting. | Requires medication for their baseline medical condition(s). Needs to be administered in a healthcare setting. | Requires medication for their baseline medical condition(s). Currently requiring a hospitalization to receive medication (e.g. hospital, hospice). | Requires medication for their baseline medical condition(s). Currently hospitalized to receive medication (e.g. hospital, hospice). |
|  |  |  |  |  |  |  | Has a central line at home (e.g. PICC line, Port-o-Cath, Broviac, Gamcath). |

**MOBILITY STATUS ASSESSMENT**

| **ORIGINAL** | **FORWARD** | **BACKWARD** | **RECONCILIATION** | **EXPERT PANEL #1** | **EXPERT PANEL #2** | **EXPERT PANEL #3** | **END-USERS** |
| --- | --- | --- | --- | --- | --- | --- | --- |
| Motilità autonoma, adeguata ad età e stadio evolutivo. | Moves independently and appropriately for age. | Si muove autonomamente e in modo appropriato per l'età. | Moves independently and appropriately for age. | Moves independently and appropriately for age. | Moves independently and appropriately for age. | Moves independently and appropriately for age. | Moves ~~independently and~~ appropriately for age. |
| Mantenimento della posizione eretta (in relazione ad età e stadio evolutivo) con assistenza e supporto. | Capable of maintaining an upright posture but requires assistance and support (inappropriate for age). | In grado di mantenere una postura eretta ma richiede assistenza e supporto (inappropriato per l'età). | Capable of maintaining an upright posture but requires assistance and support (inappropriate for age). | Capable of maintaining an upright posture but requires assistance and support (inappropriate for age). | Capable of maintaining an upright posture but requires assistance and support (inappropriate for age). | Capable of maintaining an upright posture but requires assistance and support (inappropriate for age). | ~~Capable of maintaining an upright posture~~ Can sit and stand, but requires assistance and support (inappropriate for age). |
| Incapacità di mantenimento della stazione eretta ma in grado di aiutarsi negli spostamenti. | Unable to maintain an upright posture, but capable of assisting with movements and/or changes in position. | Incapace di mantenere una postura eretta, ma in grado di assistere nei movimenti e/o nei cambi di posizione. | Unable to maintain an upright posture, but capable of assisting with movements and/or changes in position. | Unable to maintain an upright posture, but capable of assisting with movements and/or changes in position. | Unable to maintain an upright posture, but capable of assisting with movements and/or changes in position. | Unable to maintain an upright posture, but capable of assisting with movements and/or changes in position. | Unable to sit and stand, ~~to maintain an upright posture,~~ but capable of assisting with movements and/or changes in position (e.g. turning in the bed). |
| Tono muscolare ridotto che interferisce con l’equilibrio e/o il movimento. | Reduced muscle tone that interferes with balance and/or movements. | Tonicità muscolare ridotta che interferisce con l'equilibrio e/o i movimenti. | Reduced muscle tone that interferes with balance and/or movements. | ~~Reduced muscle tone that interferes with balance and/or movements.~~ |  |  |  |
| Incapacità di muoversi in modo adeguato allo stadio evolutivo. Mantenuto in carrozzina o a letto, necessita di aiuto per cambiare posizione. | Unable to move appropriately for the developmental stage. Confined to a chair or a bed, and requires total assistance to change position. | Incapace di muoversi in modo appropriato per lo stadio di sviluppo. Confina a una sedia o a un letto e richiede assistenza totale per cambiare posizione. | Unable to move appropriately for the developmental stage. Confined to a chair or a bed, and requires total assistance to change position. | Unable to move appropriately for the developmental stage. Confined to a chair or a bed, and requires total assistance to change position. | Unable to move appropriately for the developmental stage. Confined to a chair or a bed, and requires total assistance to change position. | Unable to move appropriately for the developmental stage. Confined to a chair or a bed, and requires total assistance to change position. | ~~Unable to move appropriately for the developmental stage.~~ Confined to a chair or a bed, and requires total assistance for all care needs (inappropriate for age). |
| Elevato rischio di caduta (da inciampo...) o rischio di lesioni da iperattività che richiede un una supervisione continua. | At high risk of falling (from stumbling) or injuries from hyperactivity requiring continuous supervision. | Alto rischio di cadute (dovute a inciampamenti) o lesioni da iperattività che richiedono una supervisione continua. | At high risk of falling (from stumbling) or injuries from hyperactivity requiring continuous supervision. | At high risk of falling (from stumbling) or injuries from hyperactivity requiring continuous supervision. | At high risk of falling (from stumbling) or injuries from hyperactivity requiring continuous supervision. | At high risk of falling (from stumbling) or injuries from hyperactivity requiring continuous supervision. | At high risk of falling (from stumbling) or injuries ~~from hyperactivity~~ requiring continuous supervision. |
| Contrazioni involontarie che mettono a rischio il bambino e i care givers (es. **distonie**). | Involuntary contractions that pose a risk to the child and caregivers (e.g., dystonia). | Contrazioni involontarie che rappresentano un rischio per il bambino e i caregiver (ad esempio, distonia). | Involuntary contractions that pose a risk to the child and caregivers (e.g., dystonia). | Involuntary contractions that pose a risk to the child and caregivers (e.g. dystonia). | Involuntary contractions that pose a risk to the child and caregivers (e.g. dystonia). | Involuntary contractions that pose a risk to the child ~~and caregivers~~ (e.g. dystonia). | Involuntary contractions that pose a risk to the child (e.g. dystonia). |

**COMMUNICATION ASSESSMENT**

| **ORIGINAL** | **FORWARD** | **BACKWARD** | **RECONCILIATION** | **EXPERT PANEL #1** | **EXPERT PANEL #2** | **EXPERT PANEL #3** | **END-USERS** |
| --- | --- | --- | --- | --- | --- | --- | --- |
| Comunicazione verbale e non verbale adeguata allo stadio evolutivo. | Verbal and non-verbal communication is adequate for their developmental stage. | La comunicazione verbale e non verbale è adeguata per lo stadio di sviluppo. | Verbal and non-verbal communication is adequate for their developmental stage. | Verbal and non-verbal communication is adequate for their developmental stage. | Verbal and non-verbal communication is adequate for their developmental stage. | Verbal and non-verbal communication is adequate for developmental stage. | Verbal and non-verbal communication is adequate for developmental stage. |
| Buona comprensione con necessità di illustrazione /traduzione dei termini; ritardo nell’espressione del linguaggio. | Understands well but requires some support (e.g. illustration/translation of some terms); Presents some difficulty in expressive language. | Comprende bene ma richiede un certo supporto (ad esempio, illustrazione/traduzione di alcuni termini); presenta qualche difficoltà nel linguaggio espressivo. | Understands well but requires some support (e.g. illustration/translation of some terms); Presents some difficulty in expressive language. | Understands well but requires some support (e.g. illustration/translation of some terms). ~~Presents some difficulty with expressive language.~~ | Understands well but requires some support (e.g. illustration/translation of some terms). | Understands well but requires some support (e.g. illustration or translation of some terms). | Understands well but requires some support (e.g. illustration or translation of some terms). |
| Buona comprensione con difficoltà nel linguaggio: necessita di care givers abilitato alla comprensione dei bisogni tramite anche il non verbale, legati alla familiarità con il bambino. | Understands well but requires some support (e.g. illustration/translation of some terms); Presents some difficulty in expressive language. | Comprende bene ma presenta difficoltà nel linguaggio espressivo (ad esempio, disartria, vocabolario limitato); è richiesto un caregiver familiare per assistere nella comprensione dei bisogni del bambino (ad esempio, comunicazione non verbale). | Understands well but presents difficulty with expressive language (e.g. dysarthria, limited vocabulary); a familiar caregiver is required to assist in understanding the child’s needs (e.g. non-verbal communication). | ~~Understands well but~~ Presents difficulty with expressive language (e.g. dysarthria, limited vocabulary). A familiar caregiver is required to assist in understanding the child’s needs (e.g. through non-verbal communication). | Presents difficulty with expressive language (e.g. dysarthria, limited vocabulary). A familiar caregiver is required to assist in understanding the child’s needs (e.g. through non-verbal communication). | Presents difficulty with expressive language (e.g. dysarthria, limited vocabulary). A familiar caregiver is required to assist in understanding the child’s needs (e.g. through non-verbal communication). | Presents difficulty with expressive language (e.g. dysarthria, limited vocabulary). A familiar caregiver is required to assist in understanding the child’s needs (e.g. through non-verbal communication). |
| La comunicazione è difficile da capire o interpretare. | Communication is difficult to understand and interpret for all. | La comunicazione è difficile da comprendere e interpretare per tutti. | Communication is difficult to understand and interpret for all. | ~~Communication is difficult to understand and interpret for all.~~ |  |  |  |
| Uso di metodi comunicativi diversi (es ~~linguaggio dei segni,~~ comunicatori) per bambino con deficit sensoriali. | Presence of sensory deficits that significantly impair communication, require multiple communication aids or specialized interventions. | Presenza di deficit sensoriali che compromettono significativamente la comunicazione, richiedono molteplici ausili comunicativi o interventi specializzati. | Presence of sensory deficits that significantly impair communication, require multiple communication aids or specialized interventions. | Presents of vision/hearing deficits that significantly impair communication, require multiple communication aids or specialized interventions (e.g. blindness, deafness). | Presents of vision/hearing deficits that significantly impair communication, require multiple communication aids or specialized interventions (e.g. blindness, deafness). | Presents vision/hearing deficits that significantly impair communication (e.g. blindness, deafness) and requires multiple communication aids or specialized interventions. | Presence of vision/hearing deficits (e.g. blindness, deafness) that significantly impair communication requiring ~~multiple~~ communication aids or specialized interventions (e.g. speech therapist). |
|  | Vocalizations without obvious signification or non-verbal with or without expressive language. | Vocalizzazioni prive di significato evidente o non verbali con o senza linguaggio espressivo. (new- to consider adding) | Vocalizations without obvious meaning or non-verbal with or without expressive language. | ~~Vocalizations without obvious meaning or~~ Non-verbal with or without expressive language. | Non-verbal with or without expressive language. | Non-verbal with or without expressive language. | Non-verbal (with or without expressive language). |

**SLEEP AND REST ASSESSMENT**

| **ORIGINAL** | **FORWARD** | **BACKWARD** | **RECONCILIATION** | **EXPERT PANEL #1** | **EXPERT PANEL #2** | **EXPERT PANEL #3** | **END-USERS** |
| --- | --- | --- | --- | --- | --- | --- | --- |
| Ritmo sonno veglia adeguato alle età. | Sleep-wake cycle appropriate for their age. | Ciclo sonno-veglia appropriato per l'età. | Sleep-wake cycle appropriate for their age. | Sleep-wake cycle appropriate for age. | Sleep-wake cycle appropriate for age. | Sleep-wake cycle appropriate for age. | Sleep-wake cycle appropriate for age. |
| Difficoltà nello stabilire un’adeguata routine nel ritmo sonno veglia con ricadute sulla famiglia. | Difficulty in establishing adequate sleep/wake cycle also affecting the family members. | Difficoltà nell'instaurare un adeguato ciclo sonno-veglia che influisce anche sui membri della famiglia. | Difficulty in establishing adequate sleep/wake cycle also affecting the family members. | Difficulty establishing an adequate sleep/wake cycle (e.g. difficulty in falling asleep at night resulting in daytime sleep). May responds to simple non-pharmacological interventions (e.g. soothing sleeping environment). ~~also affecting the family members.~~ | Difficulty establishing an adequate sleep/wake cycle (e.g. difficulty in falling asleep at night resulting in daytime sleep). May responds to simple non-pharmacological interventions (e.g. soothing sleeping environment). | Difficulty establishing an adequate sleep/wake cycle (e.g. difficulty in falling asleep at night resulting in daytime sleep). May responds to simple non-pharmacological interventions (e.g. soothing sleeping environment). | Difficulty establishing an adequate sleep/wake cycle (e.g. difficulty in falling asleep at night resulting in daytime sleep). May responds to ~~simple~~ non-pharmacological interventions (e.g. soothing sleeping environment). |
| Difficoltà di mantenimento del ritmo sonno veglia del bambino e/o dei genitori / care givers,per frequenti bisogni assistenziali (es. mobilizzazione, aspirazione, alimentazione...). | Difficulty maintaining an adequate sleep/wake cycle for the child and/or caregivers due to the frequent care needs (e.g., mobilization, suctioning, feeding). | Difficoltà nel mantenere un adeguato ciclo sonno-veglia per il bambino e/o i caregiver a causa delle frequenti esigenze di assistenza (ad esempio, mobilizzazione, aspirazione, alimentazione). | Difficulty maintaining an adequate sleep/wake cycle for the child and/or caregivers due to the frequent care needs (e.g., mobilization, suctioning, feeding). | Disrupted sleep/wake cycle due to routine care needs (e.g. positioning, feeding) but still fairly able to meet age-appropriate sleeping time requirement. | Disrupted sleep/wake cycle due to routine care needs (e.g. positioning, feeding) but still fairly able to meet age-appropriate sleeping time requirement. | Disrupted sleep/wake cycle due to routine care needs (e.g. positioning, feeding) but still fairly able to meet age-appropriate sleeping time requirement. | Disrupted sleep/wake cycle due to frequent routine care needs (e.g. positioning, feeding) but still fairly able to meet age-appropriate sleeping time requirement. |
| Estrema privazione di sonno di bambino e/o di genitori / care givers. | Serious sleep deprivation experienced by child and/or caregivers. | Grave privazione del sonno sperimentata dal bambino e/o dai caregiver. | Serious sleep deprivation experienced by child and/or caregivers. | Serious sleep deprivation experienced by child due to physiological or psychological reasons. May require investigations and specialized interventions. | Serious sleep deprivation experienced by child due to physiological or psychological reasons. May require investigations and specialized interventions. | Serious sleep deprivation experienced by child due to physiological or psychological reasons. May require investigations and specialized interventions. | Serious sleep deprivation experienced by child due to physical or psychological reasons. May require investigations and specialized interventions. |
| Durante il sonno sono presenti episodi che necessitano di assistenza immediata: apnea, convulsioni, inalazione che richieda aspirazione... | Potential serious health risks during sleep are present and may require immediate intervention(s) (e.g., apnea, seizures, secretions requiring suction). | Sono presenti potenziali gravi rischi per la salute durante il sonno e potrebbero richiedere interventi immediati (ad esempio, apnee, crisi convulsive, secrezioni che richiedono aspirazione). | Potential serious health risks during sleep are present and may require immediate intervention(s) (e.g., apnea, seizures, secretions requiring suction). | Potential serious health risks during sleep are present and may require immediate intervention(s) (e.g., apnea, seizures, secretions requiring suction). | ~~Serious health risks during sleep are present and require immediate intervention(s) (e.g. apnea, seizures, secretions requiring suctioning).~~ |  |  |

**CONTINENCE AND ELIMINATION ASSESSMENT**

| **ORIGINAL** | **FORWARD** | **BACKWARD** | **RECONCILIATION** | **EXPERT PANEL #1** | **EXPERT PANEL #2** | **EXPERT PANEL #3** | **END-USERS** |
| --- | --- | --- | --- | --- | --- | --- | --- |
| Continenza di urine e feci ~~(appropriate per età e grado di sviluppo).~~ | Continent of urine and feces. | Continente per l'urina e le feci. | Continent of urine and feces. | Continent of urine and feces. | Continent of urine and feces. | Continent of urine and feces. | Continent of urine and stool. |
| La cura della continenza di feci e urine è appropriato all’età ma necessita di aiuto; L’incontinenza di urine e di feci è gestita attraverso farmaci, clisteri occasionali, pannolini, guaine peniene, etc. | Urine and/or feces incontinence requiring assistance but appropriate for age (e.g., infant).  Urinary and/or feces incontinence that require interventions such as drugs, scheduled evacuations, sanitary pads, condom catheters, etc. | Incontinenza urinaria e/o fecale che richiede assistenza ma è appropriata per l'età (ad esempio, neonati).  Incontinenza urinaria e/o fecale che richiede interventi come farmaci, evacuazioni programmate, assorbenti igienici, cateteri a condoma, ecc. | Urine and/or feces incontinence requiring assistance but appropriate for age (e.g., infant).  Urinary and/or feces incontinence that require interventions such as drugs, scheduled evacuations, sanitary pads, condom catheters, etc. | Urine and/or feces incontinence requiring assistance but appropriate for age (e.g. infant, toddler). | Urine and/or feces incontinence requiring assistance but appropriate for age (e.g. infant, toddler). | Urine and/or feces incontinence requiring assistance but appropriate for age (e.g. infant, toddler). | Urine and/or stool incontinence (appropriate or not for age). May require interventions such as laxatives, scheduled evacuations, sanitary pads, condom catheter. |
|  |  |  |  | Urinary and/or feces incontinence inappropriate for age and requiring interventions such as laxatives, scheduled evacuations, sanitary pads, condom catheter. | Urinary and/or feces incontinence inappropriate for age and requiring interventions such as laxatives, scheduled evacuations, sanitary pads, condom catheter. | Urinary and/or feces incontinence inappropriate for age and requiring interventions such as laxatives, scheduled evacuations, sanitary pads, condom catheter. |  |
| ~~Incontinenza fecale o urinaria anche occasionale o~~ portatore di una stomia. | Has a stoma without issues. | Ha una stomia senza problemi. | Has a stoma without issues. | Has a stoma without issues (e.g. colostomy). | Has a stoma without issues (e.g. colostomy). | Has a stoma without issues (e.g. colostomy). | Has a functional stoma (e.g. colostomy). |
| Necessità di **cateterismi** urinari, o **clisteri quotidiani.** | Requires urinary catheterizations (intermittent or permanent catheters) and/or daily enemas. | Richiede cateterizzazioni urinarie (cateteri intermittenti o permanenti) e/o clisteri giornalieri. | Requires urinary catheterizations (intermittent or permanent catheters) and/or daily enemas. | Requires urinary catheterizations (intermittent or permanent catheters) and/or daily enemas/rectal washout. | Requires urinary catheterizations (intermittent or permanent catheters) and/or daily enemas/rectal washout. | Requires urinary catheterizations (intermittent or permanent catheters) and/or daily enemas/rectal washout. | Requires urinary catheterizations (intermittent or permanent catheterization) and/or daily enemas/rectal washout. |
| La cura della **continenza è problematica** e richiede un tempestivo intervento da parte di una persona competente (es. ~~globo vescicale~~, lavaggi rettali...). | Continence care is problematic and requires regular interventions by a trained caregiver (e.g., rectal washouts). | La cura della continenza è problematica e richiede interventi regolari da parte di un caregiver addestrato (ad esempio, clisteri rettali). | Continence care is problematic and requires regular interventions by a trained caregiver (e.g., rectal washouts). | ~~Continence care is problematic and requires regular interventions by a trained caregiver (e.g. rectal washouts).~~ |  |  |  |
| **Cateterizzazione o evacuazione complicata**/difficile (es. cateterismo ecoguidato, evacuazione chirurgica o ha una stomia che necessita di speciale attenzione più volte al giorno. | Complicated catheterization or evacuation (e.g., need for ultrasound-guided catheterization, surgical evacuation, stoma requiring attention several times per day). | Cateterizzazione o evacuazione complicata (ad esempio, necessità di cateterizzazione guidata da ultrasuoni, evacuazione chirurgica, stomia che richiede attenzione più volte al giorno). | Complicated catheterization or evacuation (e.g., need for ultrasound-guided catheterization, surgical evacuation, stoma requiring attention several times per day). | Complicated catheterization or evacuation (e.g. need for ultrasound-guided catheterization, surgical evacuation, stoma requiring attention several times per day). | Complicated catheterization or evacuation (e.g. need for ultrasound-guided catheterization, surgical evacuation, stoma requiring attention several times per day). | Complicated catheterization or evacuation (e.g. need for ultrasound-guided catheterization, surgical evacuation, stoma requiring attention several times per day). | Complicated catheterization or evacuation (e.g. need for ultrasound-guided catheterization, surgical evacuation, stoma requiring ~~attention several times per day~~ specialized care). |
| Dialisi peritoneale / Emodialisi. | Peritoneal dialysis, hemodialysis, or nephrostomy tube(s). | Dialisi peritoneale, emodialisi o tubo/i nefrostomico/i. | Peritoneal dialysis, hemodialysis, or nephrostomy tube(s). | Peritoneal dialysis, hemodialysis, or nephrostomy tube(s). | Peritoneal dialysis, hemodialysis, or nephrostomy tube(s). | Peritoneal dialysis, hemodialysis, or nephrostomy tube(s). | Peritoneal dialysis, hemodialysis, or nephrostomy tube(s). |

**PAIN ASSESSMENT**

| **ORIGINAL** | **FORWARD** | **BACKWARD** | **RECONCILIATION** | **EXPERT PANEL #1** | **EXPERT PANEL #2** | **EXPERT PANEL #3** | **END-USERS** |
| --- | --- | --- | --- | --- | --- | --- | --- |
| Nessun dolore evidente. | No signs of pain. | Nessun segno di dolore. | No signs of pain. | No report or signs of pain. | No report or signs of pain. | No report or signs of pain. | No report or signs of pain. |
| Episodi di dolore saltuari nelle ultime 4 settimane. | Occasional pain episodes in the last 4 weeks. | Episodi occasionali di dolore nelle ultime 4 settimane. | Occasional pain episodes in the last 4 weeks. | Occasional mild to moderate intensity pain in the last month. Relieved by non-pharmacological interventions or occasional mild analgesics. | Occasional mild to moderate intensity pain in the last month. Relieved by non-pharmacological interventions or occasional mild analgesics. | Occasional pain ~~mild to moderate intensity pain in the last month.~~ relieved by non-pharmacological interventions or mild analgesics as needed (e.g. acetaminophen, ibuprofen). | ~~Occasional~~ Pain can be relieved by non-pharmacological interventions or mild analgesics as needed (e.g. acetaminophen, ibuprofen). |
| Episodi di dolore pressoché quotidiani che richiedono ripetute somministrazioni di farmaci. | Almost daily pain episodes relieved by regular medication administration. | Episodi di dolore quasi giornalieri alleviati dalla somministrazione regolare di farmaci. | Almost daily pain episodes relieved by regular medication administration. | Frequent mild to moderate intensity pain relieved by a simple regimen of regular medication (e.g. daily baclofen). | Frequent mild to moderate intensity pain relieved by a simple regimen of regular medication (e.g. daily baclofen). | Frequent ~~mild to moderate intensity~~ pain requiring ~~simple~~ regular non-opioid medication (e.g. baclofen, gabapentin). | ~~Frequent~~ Pain can be relieved by regular non-opioid medication (e.g. baclofen, gabapentin, acetaminophen). |
| Episodi di dolore severo che comporta disturbi comportamentali (es. alterazioni ritmo sonno/veglia) e richiede una gestione del dolore complessa. | Severe pain episodes causing behavioral disturbances (e.g., wake/sleep cycle alterations), difficult to control but not requiring specialized interventions. | Episodi di dolore grave che causano disturbi comportamentali (ad esempio, alterazioni del ciclo sonno/veglia), difficili da controllare ma non richiedono interventi specializzati. | Severe pain episodes causing behavioral disturbances (e.g., wake/sleep cycle alterations), difficult to control but not requiring specialized interventions. | Moderate to severe pain requiring a combination of medication (e.g. regular gabapentin and acetaminophen or morphine as needed). | Moderate to severe pain requiring a combination of medication (e.g. regular gabapentin and acetaminophen or morphine as needed). | ~~Moderate to severe pain requiring a combination of medication (e.g. regular gabapentin and acetaminophen or morphine as needed).~~ |  |
| Necessità di Terapia antalgica specialistica. | Complex and refractory pain, requires specialized interventions (e.g., by pain/palliative care specialists). | Dolore complesso e refrattario, richiede interventi specializzati (ad esempio, da specialisti del dolore/palliativi). | Complex and refractory pain, requires specialized interventions (e.g., by pain/palliative care specialists). | Uncontrolled and complex pain causing behavioral disturbances requiring specialized interventions (e.g. Pain/Palliative Care specialists, opioid infusion, hospitalization). | Uncontrolled and complex pain causing behavioral disturbances requiring specialized interventions (e.g. Pain/Palliative Care specialists, opioid infusion, hospitalization). | Uncontrolled pain requiring regular medication, including opioids (e.g. morphine elixir). May require specialized interventions (e.g. Pain or Palliative Care specialists, opioid infusion, hospitalization). | ~~Uncontrolled~~ Pain can be relieved by ~~regular medication, including~~ opioids (e.g. morphine ~~elixir~~) with or without co-analgesics/adjuvants. May require specialized interventions due to the pain complexity/severity (e.g. Pain or Palliative Care specialists, opioid infusion, hospitalization). |

**CLINICAL INSTABILITY AND/OR POOR PROGNOSIS RISK ~~PREMATURE DEATH RISK~~ ASSESSMENT**

| **ORIGINAL** | **FORWARD** | **BACKWARD** | **RECONCILIATION** | **EXPERT PANEL #1** | **EXPERT PANEL #2** | **EXPERT PANEL #3** | **END-USERS** |
| --- | --- | --- | --- | --- | --- | --- | --- |
| Si prevede la sopravvivenza a lungo termine (nessun rischio attuale di peggioramenti e/ o di morte precoce). | Long-time survival expected (No foreseeable risk of deterioration and/or premature death). | Prevista una sopravvivenza a lungo termine (nessun rischio prevedibile di deterioramento e/o morte prematura). | Long-time survival expected (No foreseeable risk of deterioration and/or premature death). | Long-time survival expected (years). No foreseeable risk of deterioration and/or premature death. | Long-time survival expected (years). No foreseeable risk of deterioration and/or premature death. | Long-time survival expected (years). No foreseeable risk of deterioration and/or premature death. | Long-time survival expected (years). ~~No foreseeable risk of deterioration and/or premature death.~~ |
| E’ possibile che sopraggiungano eventi che possono mettere a **rischio la stabilità clinica** (peggioramento/deterioramento delle condizioni ). | Possible foreseeable event(s) that may jeopardize the clinical stability but not necessarily cause death. | Eventuali eventi prevedibili che potrebbero compromettere la stabilità clinica ma non necessariamente causare la morte. | Possible foreseeable event(s) that may jeopardize the clinical stability but not necessarily cause death. | ~~Possible~~ Foreseeable event(s) that may jeopardize the clinical stability but not necessarily cause death. | Foreseeable event(s) that may jeopardize the clinical stability but not necessarily cause death. | Clinically *stable* but foreseeable event(s) may jeopardize condition (e.g. high risk of sepsis). | ~~Clinically~~ *~~stable~~* ~~but~~ The child is at risk for several health complications that may compromise his/her condition but not necessarily cause death (e.g. high risk of aspiration pneumonia). |
| Non sarei sorpreso se questo paziente **morisse entro i 12 mesi.** | Greater uncertainty is foreseen. I would not be surprised if the child died within the next 12 months. | Si prevede una maggiore incertezza. Non sarei sorpreso se il bambino morisse nei prossimi 12 mesi. | Greater uncertainty is foreseen. I would not be surprised if the child died within the next 12 months. | Greater uncertainty is foreseen. I would not be surprised if the child died within the next 12 months. | Greater uncertainty is foreseen. I would not be surprised if the child died within the next 12 months. | Clinically *unstable* and progressing condition not responding to treatments. I would not be surprised if the child died within the next 12 months. | Greater uncertainty is foreseen. I would not be surprised if the child died within the next 12 months. |
