# Supplementary material for: Translation and multi-site validation of the ‘Pediatric Complex Care Needs Assessment Scale’ (ACCAPED) from Italian to English
Source: BMC Palliat Care. 2026 Mar 3;25:94. doi: 10.1186/s12904-026-02031-1 (PMC13067516; doi:10.1186/s12904-026-02031-1)
Supplement: Supplementary file 2 — Supplementary Material 2 [file 12904_2026_2031_MOESM2_ESM.docx]

**SUPPLEMENTARY RELATED MATERIAL**

EXPERT VALIDATION (Phase 2/3)

**CLINICAL VIGNETTE 1**

Salma, who is 3 years old, was diagnosed with a neurodegenerative disorder accompanied by significant global developmental delay. Recently, she has experienced a faster regression in several milestones. She can no longer sit for a year or grasp objects. She has never produced any vocalizations or expressive language. She also suffers from significant spasticity, which is well-controlled with medication.

Her other medical issues include a history of gastrointestinal reflux, currently managed with daily omeprazole. She has a seizure disorder with partial seizures occurring several times a week, each lasting a few minutes, and is on three anticonvulsant medications. These medications require frequent adjustments. For the past six months, she has been fed exclusively through a PEG tube, and all her medications are also administered via this route by her caregiver. She tolerates the feeding well, and her weight is adequate. Her breathing is normal on room air. There are no skin issues. She is incontinent of both urine and stool, using a diaper. She has difficulty falling asleep at night and sleeps most of the day.

**CLINICAL VIGNETTE 2**

Omar, a 6-year-old, has epidermolysis bullosa*. He lives in the city with both of his parents and has a brother who also has the disease. His mother is his primary caretaker. He also experiences asthma during respiratory infections. Currently, he is being treated for cellulitis with IV antibiotics in the hospital. He reports and shows signs of pain and anxiety during each daily dressing change.

He is experiencing significant difficulties meeting his nutritional needs, with about 75% of his requirements currently fulfilled, partly due to recent weight loss. He reports increasing pain when trying to swallow as the disease progresses. Liquid or pureed foods are easier to swallow but still cause discomfort. No dietitian or speech therapist has been involved yet. He uses the restroom independently. His breathing is normal. His communication skills are appropriate for his age. His mobility is also adequate, though recently he spends most of his time in bed watching videos. He sleeps well.

* Epidermolysis bullosa is a rare, non-curable condition that causes fragile skin. In the most severe cases, blisters may form inside the body, such as in the lining of the mouth or stomach. It often requires extensive dressing all over the body multiple times per day. There is a risk of fluid loss and recurrent sepsis or dehydration.

**CLINICAL VIGNETTE 3**

Felix, who is 3 days old, was just transferred by Medivac to a level 3 NICU (specialized) in an unstable condition. The mother's pregnancy was normal. He was diagnosed with complex cyanotic heart disease (hypoplastic left heart syndrome). He requires continuous oxygen at 2 L/min via nasal cannula and desaturates below 80% within a few minutes without it. He is on exclusive NGT feeding with 50% of his requirements delivered due to his respiratory status. He is irritable, cries, and does not settle down with touch. He experiences very short sleep periods because of the need for frequent nursing care, such as suctioning to prevent aspiration.

**CLINICAL VIGNETTE 4**

Marcus, 5 years old, has been diagnosed with Walker-Warburg syndrome. He has dysmorphic features, notably eye abnormalities. He has congenital hydrocephalus and has undergone ventriculo-peritoneal shunt (VP) and external ventricular drain (EVD), both of which are now removed. He has a seizure disorder; his last seizure was three weeks ago and lasted a few minutes. He takes two regular anticonvulsant medications. He is bedridden as a baseline, unable to sit even with support, and displays some spasticity. He is incontinent and uses a diaper. He sleeps fairly well.

He has frequent drooling that requires regular suctioning and glycopyrrolate. He is on strict enteral feeding (G-tube), tolerates it well, although he has a small weight for his age. Medication is administered through the G-tube by his father. He experiences regular episodes of agitation several times per week, during which he starts to cry and is not calmed by touch or parental reassurance. It is unclear if this is pain. He is not on any analgesics.

Regarding communication, he looks around, smiles occasionally when caregivers touch his face, and makes some sounds but does not speak words.

**CLINICAL VIGNETTE 5**

Fatima, 15 years old, has end-stage renal disease (ESRD). She lives with both of her parents and has four healthy siblings aged between 5 and 15 years. She also has a private caretaker at home. She has been on daily peritoneal dialysis (10 hours daily, 8 cycles) for the past 4 years. Additionally, she suffers from hypertension and takes three antihypertensive drugs (labetolol, amlodipine, enalapril) daily. Her blood pressure remains within acceptable limits, and she takes her medications without issues. She has chronic anemia related to her primary condition, which is well-controlled with regular darbopoietin. She sleeps well and has no skin issues.

She has no issues with breathing, nutrition, mobility, or communication. She experiences no uncomfortable symptoms.
